# In the Right Place at the Right Time: Habitat Representation in Protected Areas of South American *Nothofagus*-Dominated Plants after a Dispersal Constrained Climate Change Scenario

**DOI:** 10.1371/journal.pone.0119952

**Published:** 2015-03-18

**Authors:** Diego Alarcón, Lohengrin A. Cavieres

**Affiliations:** 1 Departamento de Botánica, Universidad de Concepción, Concepción, Chile; 2 Instituto de Ecología y Biodiversidad, Chile; Oklahoma State University, UNITED STATES

## Abstract

In order to assess the effects of climate change in temperate rainforest plants in southern South America in terms of habitat size, representation in protected areas, considering also if the expected impacts are similar for dominant trees and understory plant species, we used niche modeling constrained by species migration on 118 plant species, considering two groups of dominant trees and two groups of understory ferns. Representation in protected areas included Chilean national protected areas, private protected areas, and priority areas planned for future reserves, with two thresholds for minimum representation at the country level: 10% and 17%. With a 10% representation threshold, national protected areas currently represent only 50% of the assessed species. Private reserves are important since they increase up to 66% the species representation level. Besides, 97% of the evaluated species may achieve the minimum representation target only if the proposed priority areas were included. With the climate change scenario representation levels slightly increase to 53%, 69%, and 99%, respectively, to the categories previously mentioned. Thus, the current location of all the representation categories is useful for overcoming climate change by 2050. Climate change impacts on habitat size and representation of dominant trees in protected areas are not applicable to understory plants, highlighting the importance of assessing these effects with a larger number of species. Although climate change will modify the habitat size of plant species in South American temperate rainforests, it will have no significant impact in terms of the number of species adequately represented in Chile, where the implementation of the proposed reserves is vital to accomplish the present and future minimum representation. Our results also show the importance of using migration dispersal constraints to develop more realistic future habitat maps from climate change predictions.

## Introduction

In situ conservation of species, communities or ecosystems is widely recognized as the basis for effective biodiversity conservation [1]. The systematic conservation planning (sensu [2]) considers measurable conservation goals in which the representation of biodiversity in reserves is one of the most important issues [3]. Here, representation is understood as the proportion of occurrence of a conservation target within a set of protected areas [4], considering species populations, communities or ecosystems within a geographical context [5].

The Convention on Biological Diversity and other worldwide initiatives recommended a minimum representation level of ecosystems and habitats between 10% and 12% [6,7]. After the Nagoya Summit in 2010, the aim for representation in protected areas (PA) was increased to 17% for terrestrial ecosystems [8,9]. Since the implementation of these policies is a national scale issue, biases and edge artifacts have been found for ecosystems or species distributed in several countries, leading to inefficiencies when establishing protected areas at a continental scale [9]. Furthermore, the long-term persistence of current representation levels of species or ecosystems may be threatened by human-derived global changes, of which climate change is considered one of the most important [10]. As changes become more evident, understanding their potential impact on natural ecosystems will turn to be increasingly important [11], particularly how the representation level of key biodiversity habitats will be affected by projected climate conditions under different conservation schemes.

**Fig 1:**
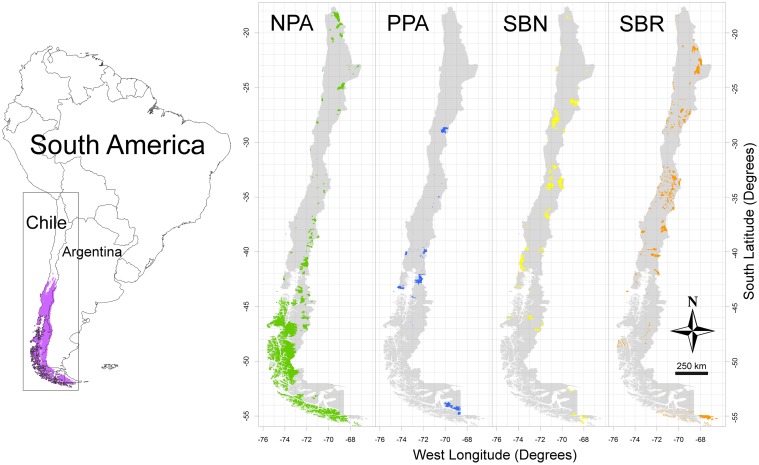
Location of *Nothofagus*-dominated temperate rainforests in South America and the geographical distribution of protected areas in Chile: national system of protected areas (NPA), private protected areas (PPA), prioritized sites for biodiversity conservation at national level (SBN) and proposed sites for biodiversity conservation at each Chilean regional administration level (SBR).

The temperate rainforests of southern South America are located in central and southern Chile with a lesser extent in neighboring areas in southwest Argentina [12], see Fig. 1. They are considered one of the most threatened biodiversity hotspots [13], including different forest communities with high levels of endemism, most of which are dominated by the genus *Nothofagus* trees [14,15]. Representation assessments have not been carried out for this ecosystem at the plant species level, while proper thresholds for them should consider the national conservation goal of 10% [16] or the Nagoya Summit suggestion of 17% [8].

Since southern South American temperate rainforests are mainly located in Chile, the *Nothofagus*-dominated temperate rainforest plants constitute a good case study for assessing the representation of the PA network at a species level, minimizing the biases found by [9] in their representation assessments for species and ecosystems located in several countries. These rainforests have several dominant tree species coexisting with a rich understory including ground and epiphytic plants, allowing comparisons among different plant groups in terms of climate change effects on their habitats and representation in PA as well.

The official Chilean national system of protected areas (NPA) includes national parks and reserves covering more than 18% of the country [17]. However, the NPA shows a strong geographical bias towards the south of the country and high altitudes in the Andes [18] see Fig. 1, and it has been considered insufficient to achieve minimum objectives for conservation at ecosystem or community scales [19] and at the species level for vertebrates [20]. Besides NPA, there are privately owned reserves (PPA), for which a legal regulation is still being developed [21]. Moreover, the Chilean national biodiversity strategy [16] proposed in 2005 a set of prioritized sites for biodiversity conservation in order to strengthen their protection. A new legislation for protected areas and biodiversity issues is currently under debate and from the former set, only a subset is now considered as priority sites for biodiversity conservation at the national level (SBN). The rest of the formerly prioritized sites are now regarded as biodiversity conservation sites at the Chilean regional administration level (SBR) [16,22], see their location in Fig. 1). However, none of the two categories of priority sites have yet been implemented, and there are no comprehensive assessments on how they could help to accomplish conservation goals both currently and in the future. Further, it is known that conclusions about representation levels of protected areas change with the target species or ecosystems [20,23]. Thus, proper evaluations of present and future representation levels should consider assessments with multiple conservation targets (i.e. species).

Niche modeling is the most commonly used method for species distribution projections [11,24,25]. As a set of probabilistic analyses that uses statistical methods linked to geographic information systems, niche modeling depicts the relationships among species spatial distributions and a series of biotic and abiotic variables related to those spatial distributions [26,27,28]. Niche modeling generates species distribution maps, allowing species distributions to be compared at different times if habitat conditions were to change. This has led to a fruitful discussion about the expected spatial shifts in species distribution due to climate change. Several studies in plants have found that species habitats will move towards higher latitudes or altitudes as a result of climate change [24,29]. Interestingly, this has started to be corroborated in the field [30,31].

However, most of potential future distribution maps produced through niche modeling methods have included uncertainties regarding the capability of species populations to effectively migrate to new territories and become part of the species habitat in this new timeframe [32–40]. Depending on the species, full-migration scenarios might be unrealistic because of biological limits for propagule dispersal across landscapes with spatial barriers, and the effective availability of new territories due to increasing human land use changes [41,42]. Different tools have been developed to take these migration constraints on species into account, and hence produce more realistic future distributions for expected climate change scenarios [35,40].

Here we took advantage of modern tools for species distribution models and assessed the representation potential of Chilean protected areas (NPA and PPA) and proposed sites (SBN and SBR), for plant species in southern South American temperate rainforests comparing two groups of dominant trees and two groups of understory plants, using niche modeling tools and considering climate change impact under a future migration constrained scenario.

## Materials and Methods

### Species selection and data sources

We selected 118 South American temperate rainforest plant species in four groups: a) *Nothofagus* tree species that dominate most of the South American temperate rainforest (n = 9); b) co-dominant tree and woody species (n = 27) from vegetation communities along with *Nothofagus*, according to [43]; c) ground ferns, considered as understory species which share their distribution with *Nothofagus* (n = 55) and d) epiphytic fern species which grow in *Nothofagus* forests (n = 27). The nomenclatural lists are based on [14] for trees and shrubs, and [44] for ferns. Species localities for trees were taken from the Chilean national forest inventory [45], and the Universidad de Concepción Herbarium (CONC), the most complete collection of Chilean plants. The list of species and the number of valid occurrences for each species is shown in S1 Table.

### Selection of climate variables and data sources

Eight variables with the lowest correlations among them were selected from the WorldClim global climate database [46] corresponding to the present climate conditions with a 30 arc-second resolution. Four of them were related to energy constraints: a) mean diurnal temperature range; b) temperature seasonality; c) maximum temperature in the warmest month and d) minimum temperature in the coldest month. The other four variables were directly related to water availability: e) annual precipitation; f) precipitation seasonality; g) precipitation in the warmest quarter and h) precipitation in the coldest quarter.

### Modeling methods and present climate models

Plant species distributions were modeled using eight techniques available through the BIOMOD R-package [47–49]: ANN for Artificial Neural Networks, CTA for Classification Tree Analysis, FDA for Flexible Discriminant Analysis, GAM for Generalized Additive Models, GBM for Generalized Boosting Models, GLM for Generalized Linear Models, MARS for Multivariate Adaptive Regression Splines, and RF for Random Forest, details of which are explained in [47] and [48]. The best models according to AUC performance values, Kappa and True skill statistics were selected for each species. We chose the model indicated as the best by a majority of the three criteria. In the few cases where they fully disagreed, we opted for AUC as the selecting criteria (see S2 Table). A current distribution map was produced considering BIOMOD cut-off thresholds to project the best specific niche model. Areas that currently correspond to human land use within these distribution maps were determined by overlaying the models produced with [45] digital information.

### Future climate data

We used future climate scenarios available from [50]. Six future scenarios were initially tested for the year 2050: cccma cgcm2 B2a, csiro mk2 B2a, hccpr hadcm3 B2a, cccma cgcm31 a1b, csiro mk30 a1b and ukmo hadcm3 a1b. The most conservative scenario was then chosen according to their least change on *Nothofagus* species distributions, which was csiro mk2 B2a. The future distribution for each species was projected using BIOMOD from the specific best niche model and the selected future climate data set.

### Migration constraints

Once future distribution maps were drawn, migration for each species was modeled using the MIGCLIM R-package [35,49]. MIGCLIM specific parameters to include dispersal kernels, potential propagule production, short-distance dispersal capacity (SDD) and probability for long-distance dispersal (LDD) were developed considering the literature available for each species [51–65] related with propagule dispersal syndromes, probable initial and optimal maturity ages and relative abundance of their populations: see S3 Table for this data. As SDD and LDD are inferred values, a sensitivity analysis of these parameters was carried out to assess how changes in their values affected the predicted habitat size for each species. For this purpose, we performed new runs of every model for each species using new SDD values accounting for 25%, 50% and 200% of the initially inferred value. For LDD, new models were run using 10%, 25%, 50%, 200%, 400% and 1000%. We compared all the MIGCLIM outputs among them and against an unrealistic scenario with no restrictions to migration, observing that the changes included in SDD and LDD values did not generate important changes in habitat sizes, whereas the full migration scenario clearly exaggerated the habitat sizes (See S4 Table and S1 Fig. for further details). Even though native forest substitution in central-south Chile reached an important magnitude during the decades of 1980s and 1990s [66–68], current assessments of land change made by the Chilean Secretary of Forests [69,70] show that this process is declining. In absence of spatial predictions for future land conversion in the whole distribution of these ecosystems, we utilized the current land use maps [44] as a conservative scenario to set the spatial barriers for future dispersion with MIGCLIM.

### Protected areas

Both present and migration constrained future distribution outputs of studied species were overlaid on maps corresponding to the following categories of Chilean protected areas: a) the national system of protected wild areas (NPA) managed by the Chilean government; b) private protected areas (PPA) managed by private owners, c) prioritized sites for biodiversity conservation at national level (SBN) as a proposal for new protected areas according to [16], and d) prioritized sites for biodiversity conservation at each Chilean regional administration level (SBR), according to [16]. Maps for the four categories were received from [22,69] and non-governmental organizations such as Así Conserva Chile and World Wildlife Fund Chile, as shown in Fig.1.

### Climate change effect assessment

Geographic information system (GIS) processes were performed using the raster R-based package [49,71]. Current and future distribution areas were compared by means of paired Student’s t-test and fitting linear models, both of which utilized the stats R-based package [49]. For assessing the climate change effect on the representation of every species habitats in protected areas, we compared the current and future representation in terms of the percentage of the habitat distribution for each time scenario. Achieving the minimum representation for each species was established according to two thresholds: 10% according to the Chilean national ecosystem representation goal for the year 2015 [16] and 17% in line with the Nagoya summit representation goal for the year 2025 [8].

## Results

### Expected effects of climate change on habitat size

**Fig 2:**
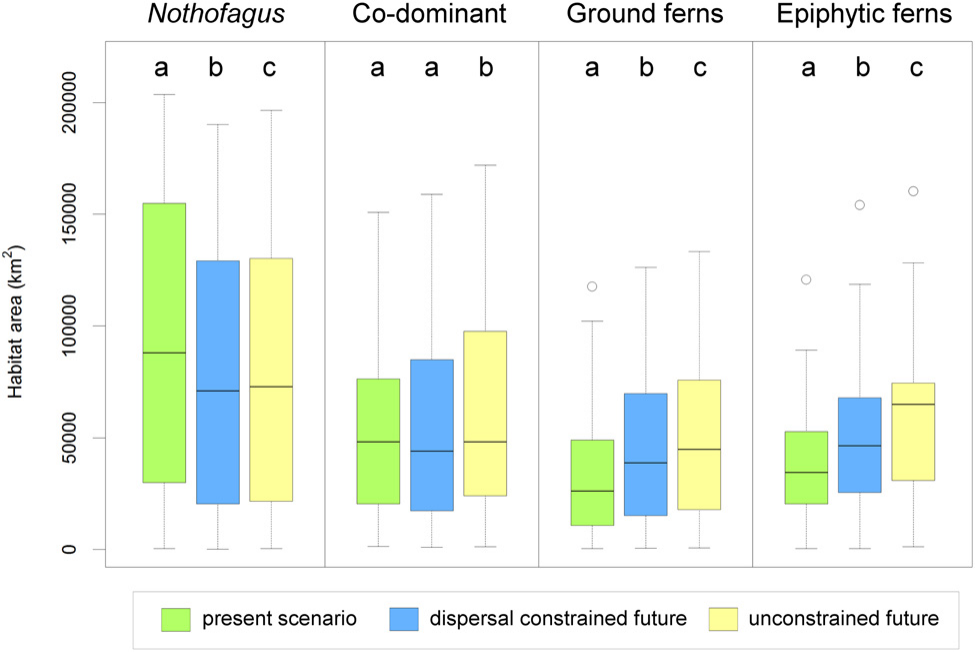
Habitat size for each species group and modeled scenario. Top letters indicate significant differences within each species group according to paired t-tests in S6 Table.

Comparisons between present and the dispersal constrained future scenario for all the studied species showed that *Nothofagus* species significantly decreased (p = 0.0047) their mean habitat size from an area of 98,553 ± 77,633 km^2^ to 87,621 ± 72,320 km^2^ in the future case (Fig. 2, and S5 and S6 Tables). For the co-dominant species group, the mean habitat area (60,130 ± 47,082 km^2^) showed no significant changes in their evaluation for the future scenario (60,095 ± 47,459 km^2^). In contrast, ground ferns habitat size (34,619 ± 28,760 km^2^) would increase in the future (45,423 ± 35,707 km^2^, p<0.0001), whereas the epiphytic ferns showed in the present scenario a mean habitat size (39,034 ± 28,910 km^2^) smaller (p<0.0001) than in the future (53,206 ± 37,833 km^2^). Thus, the future climate change scenario predicted habitat size modifications for the studied plants, with expected increases for 73.7% of the species and shrinkage for 26.3% of them (Table 1). When both unconstrained and dispersal constrained future scenarios were compared, they differed in all the species groups (p = 0.0305 in *Nothofagus* and p<0.001 in the rest of the groups, see Fig. 2 and S6 Table). Full migration scenario was significantly higher in habitat size (18.7% on average) than migration constrained models, which led us to use only the latter ones, since they consider biological bases for propagule dispersal capacity and life cycle delays, and they were very robust as indicated by the sensitivity analyses (See S4 Table).

**Table 1:** Frequencies of expected effects after climate-change scenario (%) for each species group. Features in parenthesis indicate number of species.

Effect in:		*Nothofagus*	Co-dominant	Ground ferns	Epiphytic ferns	Total species
Habitat size	Representation					
Decrease	Increase	100% (9)	51.9% (14)	7.3% (4)	11.1% (3)	25.4% (30)
Increase	Increase	-	48.1% (13)	56.4% (31)	33.3% (9)	44.9% (53)
Increase	Decrease	-	-	34.5% (19)	55.6% (15)	28.8% (34)
Decrease	Decrease	-	-	1.8% (1)	-	0.8% (1)

**Fig 3:**
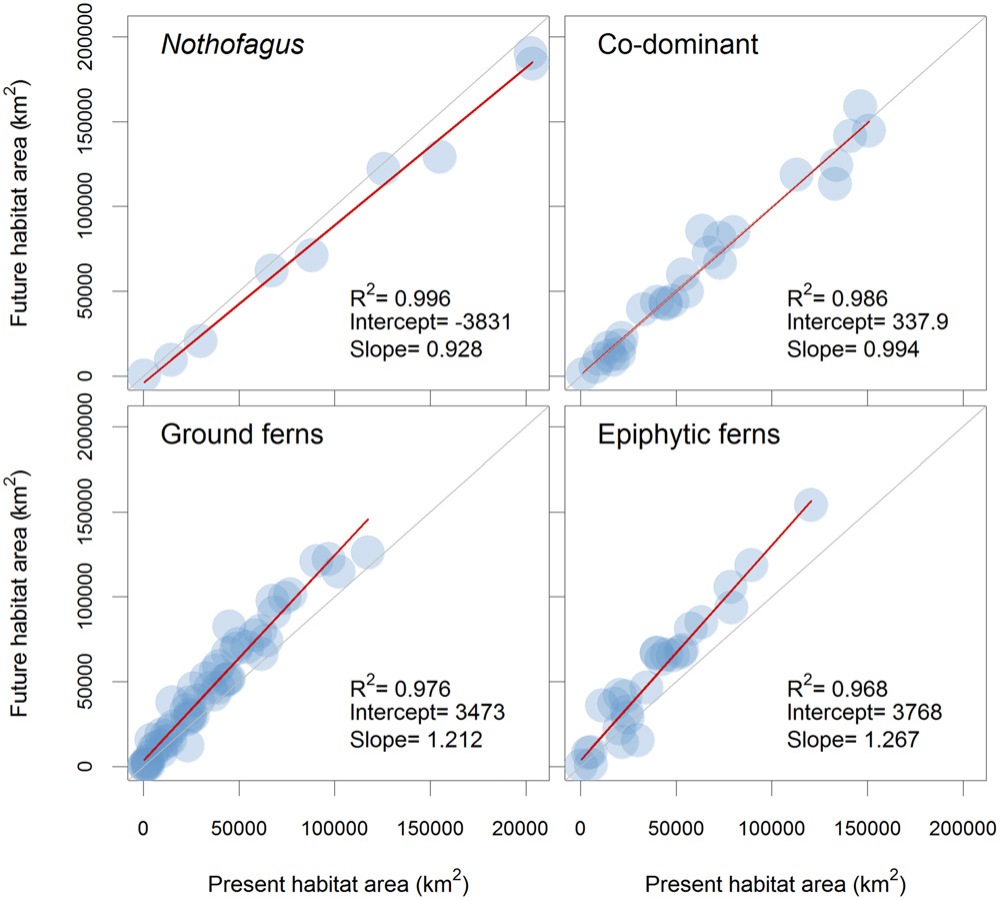
Expected changes in habitat area for all the species groups. Gray diagonal lines represent no change in habitat size; circles below the lines represent species with expected habitat size shrinkages, while circles above the lines represent species with expected habitat size increases. Red lines represent fitted linear models for each species group with their correlation, intercept and slope data.

To assess if changes in habitat size were equivalent among species irrespective of their current habitat size, we performed linear general models (LGM) between present and future habitat size for all the species groups (Fig. 3). For *Nothofagus*, the regression model slope was different (p = 0.003) and lower than 1 (0.928); furthermore, its intercept value was negative, indicating decreases in habitat size where major reductions were observed in species with larger habitats. Co-dominant species proved to maintain their habitat size, or experience only insignificant reductions, since the slope (0.994) did not differ significantly from 1 (p = 0.260). Both species groups, ground ferns and epiphytic ferns, showed linear regression slopes significantly (p<0.001) higher than 1 (1.212 and 1.267, respectively), accompanied by a positive intercept, indicating greater changes for those species with larger current habitats.

### Expected changes in species representation in protected areas

**Fig 4:**
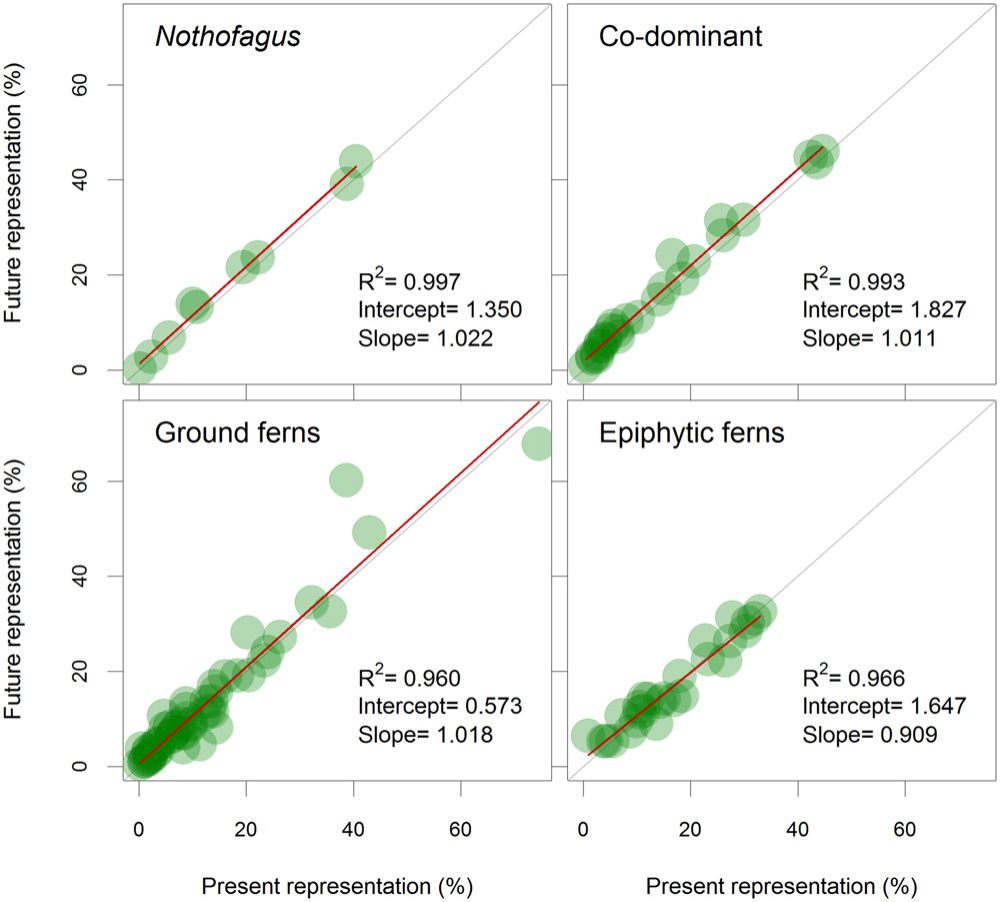
Expected changes for all the species group representation. Gray diagonal lines show no change in representation in NPA, circles under the lines represent expected shrinkages in NPA representation, while circles above the lines depict expected increases in representation in NPA. Red lines represent fitted linear models for each species group with their correlation, intercept and slope data.

For NPA areas, most of the species showed an increase in representation (70.3%) with climate change; only 29.7% of the studied species showed a decrease in their representation in NPA (Table 1). Considering species groups (Fig. 4), even though all *Nothofagus* species decreased their habitat size, their habitat representation in NPA increased. This is also seen in the fitted linear model shown in Fig. 4, where we found a positive intercept and a slope significantly (p = 0.0128) greater than 1. Likewise, even though 51.9% of the co-dominant species decreased their habitat size, all of them increased their representation in NPA, as shown by the fitted linear model with a positive intercept and a slope larger than 1 (p<0.001). On the contrary, the habitat size of most ground ferns (90.9%) and epiphytic ferns (88.9%) showed increases in the future scenario. However, future representation in NPA increased by only 63.6% for ground ferns (fitted linear model showing a positive intercept and a slope higher than 1, p = 0.035), versus a decreased future representation of 55.6% for epiphytic fern species, where the slope of the fitted linear model did not differ from 1 (p = 0.270).

**Fig 5:**
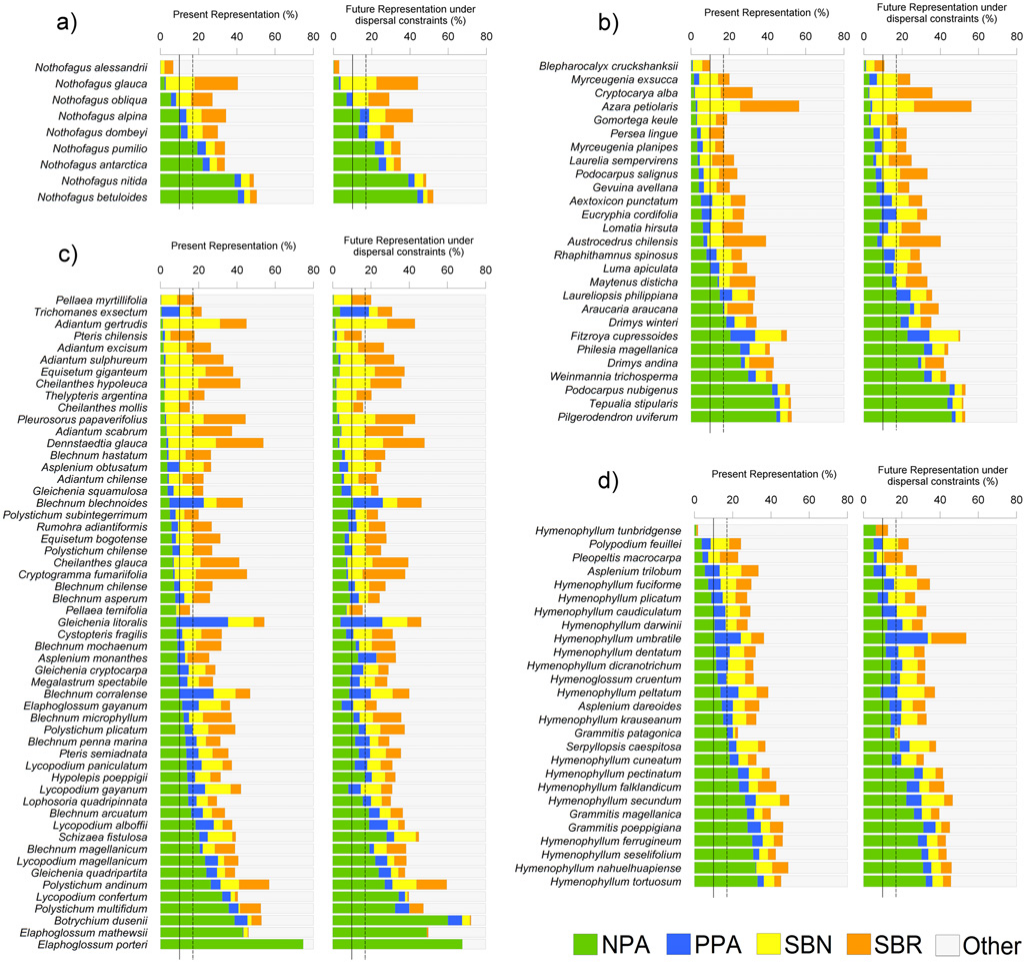
Present and future accumulated representation of a) *Nothofagus* tree species, b) co-dominant plant species, c) ground ferns and d) epiphytic ferns, in the Chilean national protected area system (NPA), private protected areas (PPA), prioritized sites for biodiversity conservation at national level (SBN) and proposed sites for biodiversity conservation at each Chilean regional administration level (SBR). Species lists in each group are hierarchically ordered by the representation of their present habitats in NPA official system. Continuous vertical lines indicate a 10% representation threshold and dotted lines indicate a 17% representation threshold.

**Fig 6:**
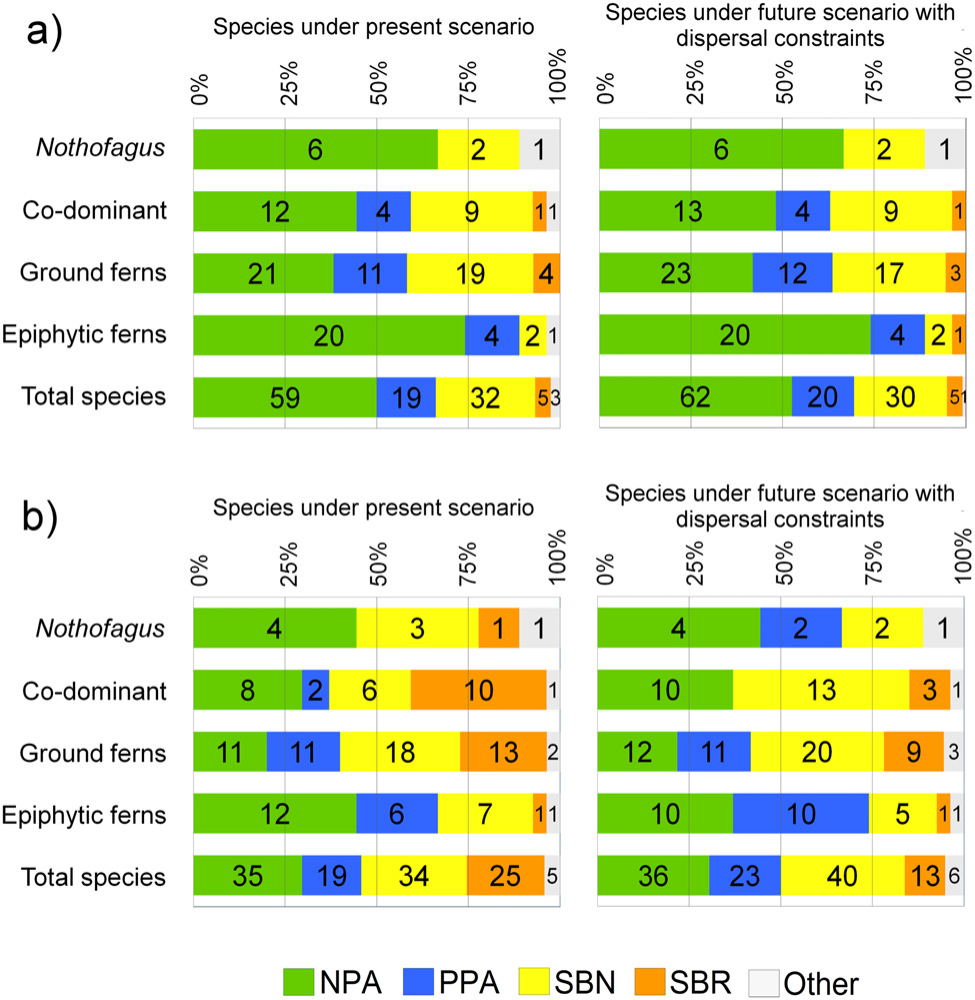
Accumulated proportion of species number with minimum representation in protected areas, considering: a) a 10% threshold as proposed by [16], and b) a 17% threshold as proposed by [8], according to Chilean national protected areas system (NPA), private protected areas (PPA), prioritized sites for biodiversity conservation at national level (SBN) and proposed sites for biodiversity conservation at each Chilean regional administration level (SBR). Features within strips indicate the aggregated number of species that achieve the minimum conservation target by each protection system.

Changes in the representation of all the species in the assessed conservation schemes can be observed in Fig. 5. When a 10% threshold was used as a minimum representation in NPA, only 50% of the total species appeared as adequately protected by the NPA official system. In particular, 67% of *Nothofagus* species (Fig. 5a), 44% of co-dominant species (Fig. 5b), 38% of ground ferns (Fig. 5c) and 74% of epiphytic ferns (Fig. 5d) are represented in NPA. The NPA representation increased in the climate change scenario for co-dominant species from 44% to 48% of adequately protected species and for ground ferns from 38% to 42%, indicating an increase from 50% to 53%, looking at all the evaluated species (Fig. 6).

Considering the 17% threshold, Chilean national protected areas provided protection for only 30% of the total number of studied species: 44% of *Nothofagus* species, 30% of co-dominant species, 20% of ground ferns and 44% of epiphytic ferns. For the future scenario, 31% of all the studied species may be well represented in the NPA, with the same species number for *Nothofagus*, but a lower species number for epiphytic ferns (37%), and an increasing representation for co-dominant plants and ground ferns up to 37% and 22%, respectively.

The inclusion of private parks (PPA) significantly affected the minimum representation for some species, such as *Nothofagus alpina* and *Nothofagus dombeyi* in the future scenario when considering a 17% threshold, and co-dominant *Aextoxicon punctatum* and *Eucryphia cordifolia* with a 10% threshold. At least 11 species of ground ferns proved to be well represented as a result of the inclusion of PPA: *Gleichenia litoralis* for both thresholds, *Blechnum asperum* and *Megalastrum spectabile* for the 10% threshold, and *Blechnum penna-marina*, *Pteris semiadnata* and *Lycopodium paniculatum* for the 17% threshold. The addition of PPA also helped four more epiphytic fern species to be well represented, such as *Asplenium trilobum* and *Hymenophyllum plicatum* with the 10% threshold in both present and future scenarios; and for the 17% threshold, 6 and 10 species respectively for each time scenario, including species like *Hymenophyllum umbratile* and *Hymenophyllum dentatum*. The inclusion of PPA generated complementary protection to the NPA, aiding 19 more species to be well represented in the present scenario (for both the 10% and 17% thresholds) and 20 more species in the future scenario considering the 10% threshold, and 23 for the 17% threshold.

Prioritized sites for biodiversity conservation at Chilean national level (SBN) were crucial for achieving a satisfactory representation in most species. If the 10% threshold is considered and SBN were implemented at the present, 110 of the 118 evaluated species (93%) achieved enough representation while 95% of the assessed species would attain the minimum representation in the future scenario. When using 17% threshold, SBN could add up to 75% of the species as suitably represented under the present scenario and the SBN location may help to adequately represent up to 84% of the studied species.

The effect of including SBR after SBN is relevant when considering the 17% threshold, since the former could increase the species under proper representation from 75% up to 96% in the present scenario, and from 84% up to 95% for the future scenario, helping to achieve enough representation for co-dominants and ground ferns. However, when using the 17% threshold, 5 species would be currently underrepresented, and only 6 of the studied species would lack minimum representation in the future scenario. The species that would not be well represented under the 17% scenario, considering NPA and PPA, even if the SBN and SBR were implemented, include the endangered tree *Nothofagus alessandrii*, co-dominant *Blepharocalyx cruckshanksii*, ground ferns *Pteris chilensis*, *Cheilanthes mollis* and *Pellaea ternifolia* and epiphytic fern *Hymenophyllum tumbridgense*.

## Discussion

The current level of representation within the Chilean NPA at the plant species level can be considered low compared to other studies: only 50% of the species are minimally represented, compared to 100% in Western Europe and 89% in the South African Cape Region [34]. This may be due to the fact that NPA is strongly southward biased and it is not coincident with the greatest diversity of vascular plants located in the center-south of Chile [18]. Interestingly, our results showed that NPA will maintain or even increase the representation level of temperate rainforest plants in the climate change scenario by mid-century, reaching up to 52.5% of species in Chile. This contrasts with a projected decreasing representation of 94% for Western Europe plant species and 78% for species in the Cape Region, for the same 10% protection threshold [34] and with some vertebrate species in Europe which are also expected to lose representation under climate change scenarios [37,38].

The future scenario for climate change indicated changes in the habitat size of the *Nothofagus*-dominated temperate rainforest of southern South America. Nevertheless, these changes are not predicted to be similar for each plant group, nor are the changes in habitat size necessarily in line with modifications in the representation level of their habitats in official areas such as NPA. For example, while the distributional habitat sizes for *Nothofagus* species are expected to decrease in the future, their representation level is projected to increase, suggesting that NPA reserves are located in useful areas for this species group in the long-term. In addition, NPA may include areas prone to be colonized by these species in a short-term future, according to the migration models. The same observation can be made with co-dominants; the species for which habitat sizes are projected to decrease (51.9% of species number) are expected to increase their representation levels in NPA. Furthermore, all of the species for which distribution size is anticipated to expand (48.1%) are expected to increase their representation proportion as well. Examples of this may be seen in species such as *N*. *alpina* and *N*. *dombeyi*, whose northern distribution and habitats at lower altitudes of the Andes mountain range are expected to be lost due to climate change. At the same time, their future habitats are expected to include present habitats that will persist in addition to new expanding zones, both coincident with the current location of NPA reserves at higher altitudes. The same situation may occur with co-dominant trees endemic to central-southern Chile that require high water availability, like *Laurelia sempervirens* and *Persea lingue*. These species are expected to decrease their northern lowland distributions where rainfall during the growing season is projected to decrease and this is coincident with areas where NPA reserves are scarce or non-existent. In addition, these species are expected to maintain their southern distributions or expand into areas close to their southern distributions, where more NPA reserves are located.

Understory ferns are expected to differ from the two groups mentioned above: 90.9% of ground fern species are projected to expand their habitat size, but only 63.6% will most likely increase their representation level in NPA. Nonetheless, the most striking difference is seen in epiphytic ferns; 88.9% of these species are expected to gain distribution area, but only 44.4% are anticipated to expand their representation proportion. This is most likely related to the fact that NPA units are geographically biased towards higher elevations, while a significant proportion of epiphytic fern species are prone to expand to lower altitude forests not included in NPA. The latter implies that the location of NPA reserves will continue to be useful in the future, except for one third (29.7%) of the total studied species for which new colonizable areas will not coincide with NPA or some habitats currently protected by NPA may not be climatically suitable in the future. Among these species we can mention a set of ground ferns distributed in the Mediterranean climate of central Chile, such as *Cheilanthes hypoleuca*, *Dennstaedtia glauca*, *Pteris chilensis* and *Thelypteris argentina*, whose future representation is expected to decrease because the few NPA units existing in central Chile may not be useful for the conservation of these ferns after climate change. Some other ground fern species with larger habitat distributions like *Blechnum magellanicum* and *Lycopodium magellanicum* are expected to have a distribution expansion outside NPA units, resulting in a decrease in their future representation. Other cases like *Elaphoglossum porteri* or *Elaphoglossum gayanum* have very small present distribution areas, and the decrease in their representation level can be attributed to the fact that their current distributions are mostly within NPA reserves, and in the future they would expand their populations to other places outside NPA. The ecological niches of both groups ground and epiphytic ferns resulted more heterogeneous compared to those of the dominant trees, with a differential response in habitat size changes also, and hence in their representation levels in the NPA system.

The current location of NPA parks and reserves assures enough future protection to maintain the same number of species of *Nothofagus* trees and epiphytic ferns. Thus, the current location of NPA reserves is important for current representation and it will continue to be important when temperate rainforest species face the expected climate change effects in the future. This suggests that any alterations to NPA units, such as clipping or area reduction for productive exploitations or changes in their main objectives to aims other than preserving habitat representation, are therefore strongly discouraged from the standpoint of biological resource policies for overcoming climate change. Despite the high proportion of Chilean land dedicated to NPA, representation goals are still not being met when biodiversity levels are considered, such as plant species levels, and future climate change for the region. Moreover, the current role of NPA in long-term biodiversity conservation is not fully assured yet since management plans have not been completed for all their units. In recent years, there has been a debate over redefining the main objectives of the NPA system and the government has included tourism among them, leading to building facilities in formerly fully preserved lands. Restricting grazing for the livestock of nearby communities, alien species control and setting maximum visitor limits inside reserves are among the challenges to be accomplished in order to assure the NPA long-term conservation role.

PPA reserves have not yet been fully formalized under law. However, our results showed that they would have a very positive complementary effect to official NPA, since PPA would add conserved habitats for 97.5% of the evaluated species, increasing additional conservation for 73.7% of the studied species in the future scenario. When minimum representation thresholds were considered, PPA reserves were shown to help 16.1% of the studied species to attain a well-conserved status, for both thresholds in the present scenario. Moreover, they could aid 16.9% of these species to obtain a well-represented future condition under the 10% threshold; this is 19.5% of the species if the 17% threshold were used for the future scenario. The positive effects of including PPA are more important for understory fern species than dominant tree species because the distribution of PPA areas is complementary to NPA in low elevations, where some understory ferns may tend to expand their distribution in the future. Since NPA reserves are located unevenly across the Chilean territory, proportionally more distributed towards southern and higher areas, PPA may spatially fit as a significant complement for NPA areas, in the same direction of those found by other evaluations of private reserves [72,73].

As proposed for new protected areas under the Chilean national biodiversity strategy [16], the implementation of the SBN sites virtually completes the supplementary needs for minimum representation for most of the studied species after considering NPA and PPA, both in the present and future scenarios with a 10% threshold. The effect of including the SBR sites resulted important to accomplish satisfactory levels of species representation when applying the most restrictive threshold. This contrasts to the fact that Chilean environmental policy has recently considered most of these SBR areas in a low priority for being implemented as reserves, because they mostly comprised private properties. If the Nagoya summit threshold were applied, the set of species mentioned at the end of Results would still not achieve a minimum representation with NPA, PPA, SBN and SBR combined; most of them have habitats included in a Mediterranean-climate zone with scarce representation under conservation schemes, associated with high replacement due to human land-use. If SBN and SBR were carried out, it would be wise to include the habitat area of those species in the proposed reserves as well, especially for the endangered tree *Nothofagus alessandrii*, tree *Blepharocalyx cruckshanksii* and the ground fern *Pteris chilensis*.

Even though climate change is expected to affect the habitats of the evaluated plant species from these important South American temperate rainforests, the current location of the conservation units determines that there will be no significant change in terms of the number of species with a minimum representation for the evaluated future scenario. Analyzing climate change effects on habitat size and representation in parks, considering only the dominant trees as a basis may not be applicable to other species groups as it was the case for understory plants in this study. The evaluated groups are an example set of plants growing in a temperate rainforest, and their distributions are expected to react differently with climate change. Since their habitat representation may be achieved in a specific manner for every group, it is important to realize that this kind of assessment should consider a diverse and wide spectrum of species groups.

## Conclusions

When applying the future scenario, most of the dominant tree species are expected to decrease in habitat size while their representation levels increase, but most of the understory plant species increased their habitat size, as well as their representation level in NPA. Contrary to former worldwide analyses, our study indicated that official Chilean NPA reserves are spatially arranged in a way which corresponds to areas prone to be maintained or expanded as useful habitats for most of the studied species after climate change. Our results highlight the need for strengthening NPA in order to contribute to overcome the climate change in South American temperate rainforest plants. Therefore, it is extremely important not to affect or reduce NPA units due to policy changes or productive purposes.

Private contributions to conservation through PPA reserves help official NPA to achieve minimum representation of most of the studied plant species. This will remain to be true or even increase in importance after climate change for many of the studied species. Implementing the prioritized SBN as formal reserves is also strongly recommended since they virtually complete the minimum representation needs for most of the studied plants in the present as well as the future climate-change scenario if the 10% threshold is observed. Considering an aggregated implementation of proposed SBR would be essential if 17% threshold was taken into account. The evaluated species were chosen because they represent some substantial structural elements of South American temperate rainforests; dominant elements such as *Nothofagus* tree species and co-dominant woody species are expected to react in their own specific way, different from understory components such as ground ferns and epiphytic ferns. Consequently, climate change assessments must be carried out on a species level and it is important not to assume that expected effects to dominant tree species are necessarily applicable to understory species. Finally, the use of migration dispersal constraints should always be included when more realistic future habitat maps are developed.

## Supporting Information

S1 Fig Sensitivity analysis of changes in MIGCLIM parameters SDD (short distance dispersal) and LDD (probability for long distance dispersal) and their effects on the habitat area size for all the studied species, following S4 Table. Vertical red line represents 0% change in habitat size considering the parameters set in S3 Table as 100% for both SDD and LDD. A full migration scenario is also included as a comparison for habitat sizes without using MIGCLIM.
(TIF)

S1 Table Plant species list included in the assessment. Each species is presented according to taxonomic family, species group and endemism to South American temperate rainforests, and the number of valid occurrences used in niche modeling.
(DOC)

S2 Table Parameter values (AUC, Kappa and TSS) to determine the best model for each species. The following modeling techniques were included: ANN for Artificial Neural Networks, CTA for Classification Tree Analysis, FDA for Flexible Discriminant Analysis, GAM for Generalized Additive Models, GBM for Generalized Boosting Models, GLM for Generalized Linear Models, MARS for Multivariate Adaptive Regression Splines, and RF for Random Forest.
(DOC)

S3 Table Assumptions used for each species in order to set MIGCLIM parameters. (DOC)

S4 Table Sensitivity analysis of MIGCLIM parameters SDD (short distance dispersal) and LDD (probability for long distance dispersal) and their effects on the habitat area size for all the studied species: a) by species group, and b) by each species. A full migration scenario is also included as a comparison for habitat sizes without using MIGCLIM.
(DOC)

S5 Table Modeled habitat area size (km^2^) under assessed scenarios: present and future (year 2050) with modeled dispersal constraints for each species and their representation under different conservation schemes in Chile. (DOC)

S6 Table Habitat size comparisons among scenarios within species groups (obtained with paired t-tests). (DOC)
