# Supplementary figures and images for: In the Right Place at the Right Time: Habitat Representation in Protected Areas of South American Nothofagus-Dominated Plants after a Dispersal Constrained Climate Change Scenario
Source: PLoS One. 2015 Mar 18;10(3):e0119952. doi: 10.1371/journal.pone.0119952 (PMC4364909; doi:10.1371/journal.pone.0119952)

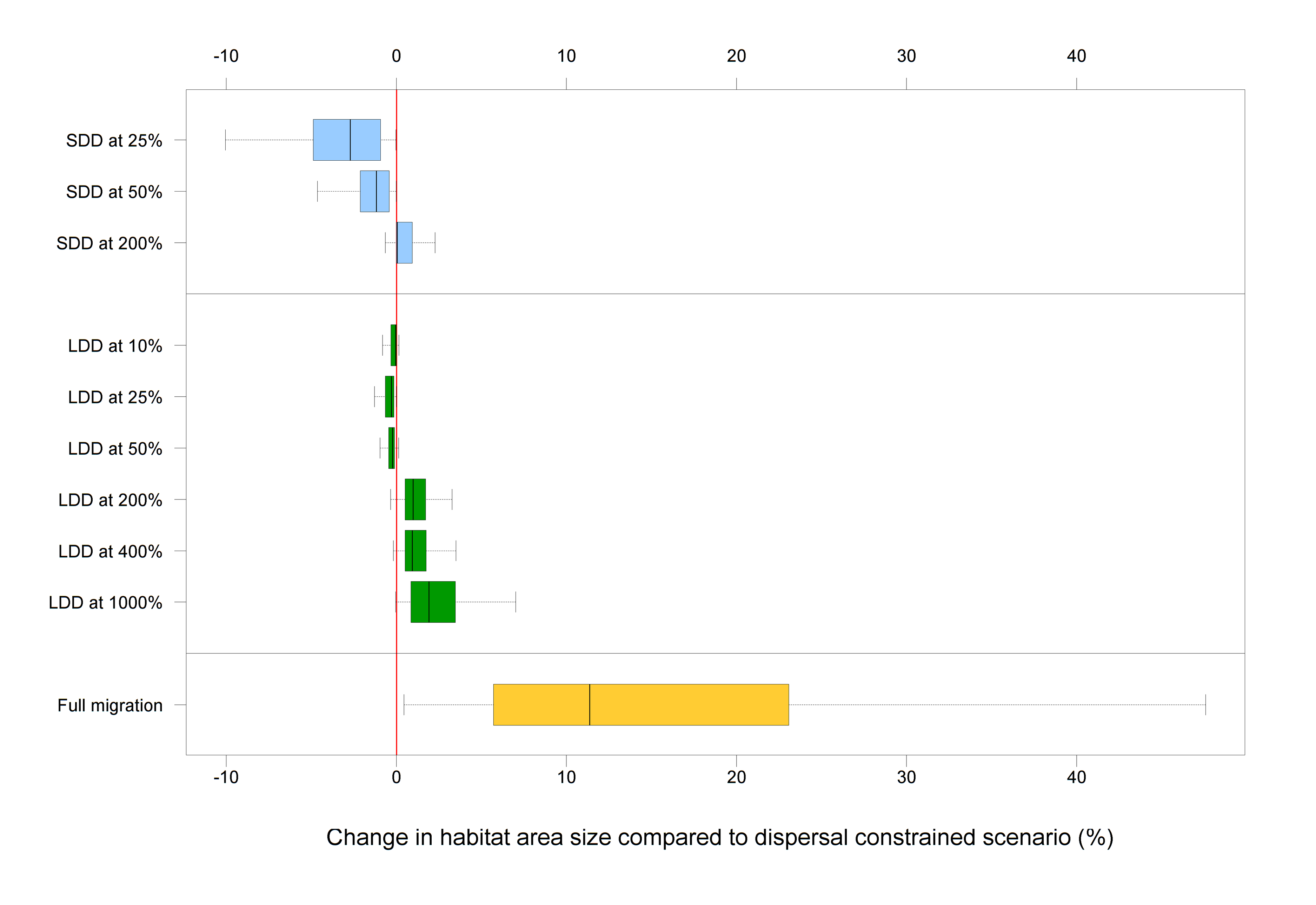

Supplement: S1 Fig — Vertical red line represents 0% change in habitat size considering the parameters set in S3 Table as 100% for both SDD and LDD. A full migration scenario is also included as a comparison for habitat sizes without using MIGCLIM. (TIF) [file pone.0119952.s001.tif]
