# Supplementary material for: In the Right Place at the Right Time: Habitat Representation in Protected Areas of South American Nothofagus-Dominated Plants after a Dispersal Constrained Climate Change Scenario
Source: PLoS One. 2015 Mar 18;10(3):e0119952. doi: 10.1371/journal.pone.0119952 (PMC4364909; doi:10.1371/journal.pone.0119952)
Supplement: S1 Table — Each species is presented according to taxonomic family, species group and endemism to South American temperate rainforests, and the number of valid occurrences used in niche modeling. (DOC) [file pone.0119952.s002.doc]

**Table S1. Plant species list included in the assessment**. Each species is presented according to taxonomic family, species group and endemism to South American temperate rainforests, and the number of valid occurrences used in niche modeling.

| Plant species | Family | Species group | Endemic to South American temperate rainforest ^a^ | Number of occurrences |
| --- | --- | --- | --- | --- |
| *Adiantum chilense* Kaulf. | Pteridaceae | Ground fern | No | 297 |
| *Adiantum excisum* Kunze | Pteridaceae | Ground fern | Yes ^b^ | 78 |
| *Adiantum gertrudis* Espinosa | Pteridaceae | Ground fern | Yes | 20 |
| *Adiantum scabrum* Kaulf. | Pteridaceae | Ground fern | Yes | 84 |
| *Adiantum sulphureum* Kaulf. | Pteridaceae | Ground fern | Yes ^b^ | 129 |
| *Aextoxicon punctatum* Ruiz & Pav. | Aextoxicaceae | Co-dominant species | Yes | 3,391 |
| *Araucaria araucana* (Molina) K. Koch | Araucariaceae | Co-dominant species | Yes | 6,946 |
| *Asplenium dareoides* Desv. | Aspleniaceae | Epiphytic fern | Yes ^b^ | 162 |
| *Asplenium monanthes* L. | Aspleniaceae | Ground fern | No | 7 |
| *Asplenium obtusatum* G. Forst. var. *sphenoides* (Kunze) C. Chr. ex Skottsb. | Aspleniaceae | Ground fern | Yes ^c^ | 44 |
| *Asplenium trilobum* Cav. | Aspleniaceae | Epiphytic fern | Yes | 49 |
| *Austrocedrus chilensis* (D. Don) Pic.Serm. & Bizzarri | Cupressaceae | Co-dominant species | Yes | 426 |
| *Azara petiolaris* (D. Don) I.M. Johnst. | Salicaceae | Co-dominant species | Yes | 73 |
| *Blechnum arcuatum* J. Remy | Blechnaceae | Ground fern | Yes | 42 |
| *Blechnum asperum* (Klotzsch) J.W. Sturm | Blechnaceae | Ground fern | Yes | 15 |
| *Blechnum blechnoides* Keyserl. | Blechnaceae | Ground fern | Yes | 30 |
| *Blechnum chilense* (Kaulf.) Mett. | Blechnaceae | Ground fern | No | 133 |
| *Blechnum corralense* Espinosa | Blechnaceae | Ground fern | Yes | 15 |
| *Blechnum hastatum* Kaulf. | Blechnaceae | Ground fern | Yes ^b^ ^c^ | 309 |
| *Blechnum magellanicum* (Desv.) Mett. | Blechnaceae | Ground fern | Yes | 111 |
| *Blechnum microphyllum* (Goldm.) C.V. Morton | Blechnaceae | Ground fern | Yes | 60 |
| *Blechnum mochaenum* G. Kunkel | Blechnaceae | Ground fern | No | 129 |
| *Blechnum penna-marina* (Poir.) Kuhn | Blechnaceae | Ground fern | No | 146 |
| *Blepharocalyx cruckshanksii* (Hook. & Arn.) Nied. | Myrtaceae | Co-dominant species | Yes | 253 |
| *Botrychium dusenii* (H. Christ) Alston | Ophioglossaceae | Ground fern | Yes | 15 |
| *Cheilanthes glauca* (Cav.) Mett. | Pteridaceae | Ground fern | Yes ^b^ | 123 |
| *Cheilanthes hypoleuca* (Kunze) Mett. | Pteridaceae | Ground fern | Yes ^b^ | 104 |
| *Cheilanthes mollis* (Kunze) C. Presl | Pteridaceae | Ground fern | No | 98 |
| *Cryptocarya alba* (Molina) Looser | Lauraceae | Co-dominant species | Yes | 11,386 |
| *Cryptogramma fumariifolia* (Phil. ex Baker) H. Christ | Pteridaceae | Ground fern | Yes ^b^ | 19 |
| *Cystopteris fragilis* (L.) Bernh. | Dryopteridaceae | Ground fern | No | 137 |
| *Dennstaedtia glauca* (Cav.) C. Chr. ex Looser | Dennstaedtiaceae | Ground fern | No | 20 |
| *Drimys andina* (Reiche) R. Rodr. & Quezada | Winteraceae | Co-dominant species | Yes | 38 |
| *Drimys winteri* J.R. Forst. & G. Forst. | Winteraceae | Co-dominant species | No | 8,543 |
| *Elaphoglossum gayanum* (Fée) T. Moore | Lomariopsidaceae | Ground fern | No | 10 |
| *Elaphoglossum mathewsii* (Fée) T. Moore | Lomariopsidaceae | Ground fern | Yes | 11 |
| *Elaphoglossum porteri* Hicken | Lomariopsidaceae | Ground fern | Yes | 5 |
| *Equisetum bogotense* Kunth | Equisetaceae | Ground fern | No | 157 |
| *Equisetum giganteum* L. | Equisetaceae | Ground fern | No | 65 |
| *Eucryphia cordifolia* Cav. | Eucryphiaceae | Co-dominant species | Yes | 3,998 |
| *Fitzroya cupressoides* (Molina) I.M. Johnst. | Cupressaceae | Co-dominant species | Yes | 6,158 |
| *Gevuina avellana* Molina | Proteaceae | Co-dominant species | Yes | 6,135 |
| *Gleichenia cryptocarpa* Hook. | Gleicheniaceae | Ground fern | Yes | 63 |
| *Gleichenia litoralis* (F. Phil.) C. Chr. | Gleicheniaceae | Ground fern | Yes | 17 |
| *Gleichenia quadripartita* (Poir.) T. Moore | Gleicheniaceae | Ground fern | Yes | 82 |
| *Gleichenia squamulosa* (Desv.) T. Moore | Gleicheniaceae | Ground fern | Yes ^c^ | 107 |
| *Gomortega keule* (Molina) Baill. | Gomortegaceae | Co-dominant species | Yes | 66 |
| *Grammitis magellanica* Desv. | Grammitidaceae | Epiphytic fern | Yes ^c^ | 62 |
| *Grammitis patagonica* (C. Chr.) Parris | Grammitidaceae | Epiphytic fern | No | 7 |
| *Grammitis poeppigiana* (Mett.) Pic.Serm. | Grammitidaceae | Epiphytic fern | No | 20 |
| *Hymenoglossum cruentum* (Cav.) C. Presl | Hymenophyllaceae | Epiphytic fern | Yes ^c^ | 70 |
| *Hymenophyllum caudiculatum* Mart. | Hymenophyllaceae | Epiphytic fern | Yes ^c^ | 94 |
| *Hymenophyllum cuneatum* Kunze | Hymenophyllaceae | Epiphytic fern | Yes ^c^ | 18 |
| *Hymenophyllum darwinii* Hook. f. ex Bosch | Hymenophyllaceae | Epiphytic fern | Yes | 20 |
| *Hymenophyllum dentatum* Cav. | Hymenophyllaceae | Epiphytic fern | Yes | 95 |
| *Hymenophyllum dicranotrichum* (C. Presl) Hook. ex Sadeb. | Hymenophyllaceae | Epiphytic fern | Yes | 42 |
| *Hymenophyllum falklandicum* Baker | Hymenophyllaceae | Epiphytic fern | Yes ^c^ | 20 |
| *Hymenophyllum ferrugineum* Colla | Hymenophyllaceae | Epiphytic fern | No | 41 |
| *Hymenophyllum fuciforme* Sw. | Hymenophyllaceae | Epiphytic fern | Yes ^c^ | 34 |
| *Hymenophyllum krauseanum* Phil. | Hymenophyllaceae | Epiphytic fern | Yes | 62 |
| *Hymenophyllum nahuelhuapiense* Diem & J.S. Licht. | Hymenophyllaceae | Epiphytic fern | Yes | 5 |
| *Hymenophyllum pectinatum* Cav. | Hymenophyllaceae | Epiphytic fern | Yes ^c^ | 89 |
| *Hymenophyllum peltatum* (Poir.) Desv. | Hymenophyllaceae | Epiphytic fern | No | 56 |
| *Hymenophyllum plicatum* Kaulf. | Hymenophyllaceae | Epiphytic fern | Yes | 62 |
| *Hymenophyllum secundum* Hook. & Grev. | Hymenophyllaceae | Epiphytic fern | Yes ^c^ | 71 |
| *Hymenophyllum seselifolium* C. Presl | Hymenophyllaceae | Epiphytic fern | No | 61 |
| *Hymenophyllum tortuosum* Hook. & Grev. | Hymenophyllaceae | Epiphytic fern | Yes ^c^ | 81 |
| *Hymenophyllum tunbrigense* (L.) Sm. | Hymenophyllaceae | Epiphytic fern | No | 15 |
| *Hymenophyllum umbratile* Diem & J.S. Licht. | Hymenophyllaceae | Epiphytic fern | Yes | 13 |
| *Hypolepis poeppigii* (Kunze) R. Rodr. | Dennstaedtiaceae | Ground fern | Yes ^b^ | 105 |
| *Laurelia sempervirens* (Ruiz & Pav.) Tul. | Monimiaceae | Co-dominant species | Yes | 635 |
| *Laureliopsis philippiana* (Looser) Schodde | Monimiaceae | Co-dominant species | Yes | 6,404 |
| *Lomatia hirsuta* (Lam.) Diels | Proteaceae | Co-dominant species | No | 1,136 |
| *Lophosoria quadripinnata* (J.F. Gmel.) C. Chr. | Dicksoniaceae | Ground fern | No | 87 |
| *Luma apiculata* (DC.) Burret | Myrtaceae | Co-dominant species | Yes | 6,713 |
| *Lycopodium alboffii* Rolleri | Lycopodiaceae | Ground fern | Yes | 18 |
| *Lycopodium confertum* Willd. | Lycopodiaceae | Ground fern | Yes | 22 |
| *Lycopodium gayanum* J. Remy | Lycopodiaceae | Ground fern | Yes ^c^ | 34 |
| *Lycopodium magellanicum* (P. Beauv.) Sw. | Lycopodiaceae | Ground fern | Yes | 104 |
| *Lycopodium paniculatum* Desv. | Lycopodiaceae | Ground fern | Yes | 69 |
| *Maytenus disticha* (Hook. f.) Urb. | Celastraceae | Co-dominant species | Yes | 52 |
| *Megalastrum spectabile* (Kaulf.) A.R. Sm. & R.C. Moran | Dryopteridaceae | Ground fern | Yes | 83 |
| *Myrceugenia planipes* (Hook. & Arn.) O. Berg | Myrtaceae | Co-dominant species | Yes | 829 |
| *Myrceugenia exsucca* (DC.) O. Berg | Myrtaceae | Co-dominant species | Yes | 113 |
| *Nothofagus alessandrii* Espinosa | Nothofagaceae | Dominant species | Yes | 64 |
| *Nothofagus alpina* (Poepp. & Endl.) Oerst. | Nothofagaceae | Dominant species | Yes | 10,759 |
| *Nothofagus antarctica* (G. Forst.) Oerst. | Nothofagaceae | Dominant species | Yes | 29,159 |
| *Nothofagus betuloides* (Mirb.) Oerst. | Nothofagaceae | Dominant species | Yes | 32,388 |
| *Nothofagus dombeyi* (Mirb.) Oerst. | Nothofagaceae | Dominant species | Yes | 27,092 |
| *Nothofagus glauca* (Phil.) Krasser | Nothofagaceae | Dominant species | Yes | 4,343 |
| *Nothofagus nitida* (Phil.) Krasser | Nothofagaceae | Dominant species | Yes | 37,581 |
| *Nothofagus obliqua* (Mirb.) Oerst. | Nothofagaceae | Dominant species | Yes ^d^ | 42,600 |
| *Nothofagus pumilio* (Poepp. & Endl.) Krasser | Nothofagaceae | Dominant species | Yes | 28,747 |
| *Pellaea myrtillifolia* Mett. ex Kuhn | Pteridaceae | Ground fern | Yes ^b^ | 22 |
| *Pellaea ternifolia* (Cav.) Link | Pteridaceae | Ground fern | No | 29 |
| *Persea lingue* (Miers ex Bertero) Nees | Lauraceae | Co-dominant species | Yes | 3,182 |
| *Philesia magellanica* J.F. Gmel. | Philesiaceae | Co-dominant species | Yes | 72 |
| *Pilgerodendron uviferum* (D. Don) Florin | Cupressaceae | Co-dominant species | Yes | 20,591 |
| *Pleopeltis macrocarpa* (Bory ex Willd.) Kaulf. | Polypodiaceae | Epiphytic fern | No | 16 |
| *Pleurosorus papaverifolius* (Kunze) Mett. | Aspleniaceae | Ground fern | Yes ^b^ | 36 |
| *Podocarpus salignus* D. Don | Podocarpaceae | Co-dominant species | Yes | 19,391 |
| *Podocarpus nubigenus* Lindl. | Podocarpaceae | Co-dominant species | Yes | 99 |
| *Polypodium feuillei* Bertero | Blechnaceae | Epiphytic fern | Yes ^b^ | 136 |
| *Polystichum andinum* Phil. | Dryopteridaceae | Ground fern | Yes | 38 |
| *Polystichum chilense* (H. Christ) Diels | Dryopteridaceae | Ground fern | Yes | 118 |
| *Polystichum multifidum* (Mett.) H. Christ | Dryopteridaceae | Ground fern | Yes | 29 |
| *Polystichum plicatum* (Poepp. ex Kunze) Hicken | Dryopteridaceae | Ground fern | Yes ^b^ | 124 |
| *Polystichum subintegerrimum* (Hook. & Arn.) R. Rodr. | Dryopteridaceae | Ground fern | Yes | 22 |
| *Pteris chilensis* Desv. | Pteridaceae | Ground fern | Yes | 33 |
| *Pteris semiadnata* Phil. | Pteridaceae | Ground fern | Yes | 38 |
| *Rhaphithamnus spinosus* (Juss.) Moldenke | Verbenaceae | Co-dominant species | No | 3,398 |
| *Rumohra adiantiformis* (G. Forst.) Ching | Dryopteridaceae | Ground fern | No | 78 |
| *Schizaea fistulosa* Labill. | Schizaeaceae | Ground fern | Yes | 18 |
| *Serpyllopsis caespitosa* (Gaudich.) C. Chr. | Hymenophyllaceae | Epiphytic fern | Yes ^c^ | 51 |
| *Tepualia stipularis* (Hook. & Arn.) Griseb. | Myrtaceae | Co-dominant species | Yes | 25,869 |
| *Thelypteris argentina* (Hieron.) Abbiatti | Thelypteridaceae | Ground fern | No | 44 |
| *Trichomanes exsectum* Kunze | Hymenophyllaceae | Ground fern | Yes | 17 |
| *Weinmannia trichosperma* Cav. | Cunoniaceae | Co-dominant species | Yes | 24,577 |

^a^ South American temperate rainforest range includes central and southern Chile and neighboring areas in Argentina.

^b^ Distribution also includes other small neighboring areas.

^c^ Distribution also includes Juan Fernández Archipelago in the Pacific Ocean.

^d^ This species includes formerly named *Nothofagus macrocarpa* (A. DC.) F.M. Vázquez & R. Rodr. populations following [74].
