# Supplementary material for: In the Right Place at the Right Time: Habitat Representation in Protected Areas of South American Nothofagus-Dominated Plants after a Dispersal Constrained Climate Change Scenario
Source: PLoS One. 2015 Mar 18;10(3):e0119952. doi: 10.1371/journal.pone.0119952 (PMC4364909; doi:10.1371/journal.pone.0119952)
Supplement: S2 Table — The following modeling techniques were included: ANN for Artificial Neural Networks, CTA for Classification Tree Analysis, FDA for Flexible Discriminant Analysis, GAM for Generalized Additive Models, GBM for Generalized Boosting Models, GLM for Generalized Linear Models, MARS for Multivariate Adaptive Regression Splines, and RF for Random Forest. (DOC) [file pone.0119952.s003.doc]

**Table S2.** **Parameter values (AUC, Kappa and TSS) to determine the best model for each species.** The following modeling techniques were included: ANN for Artificial Neural Networks, CTA for Classification Tree Analysis, FDA for Flexible Discriminant Analysis, GAM for Generalized Additive Models, GBM for Generalized Boosting Models, GLM for Generalized Linear Models, MARS for Multivariate Adaptive Regression Splines, and RF for Random Forest.

| Species | AUC | | Kappa | | TSS | | Final best model |
| --- | --- | --- | --- | --- | --- | --- | --- |
|  | Best model | Value | Best model | Value | Best model | Value |  |
| *Adiantum chilense* | RF | 0.979 | RF | 0.835 | RF | 0.902 | RF |
| *Adiantum excisum* | RF | 0.978 | RF | 0.807 | RF | 0.910 | RF |
| *Adiantum gertrudis* | GBM | 0.979 | RF | 0.722 | GBM | 0.909 | GBM |
| *Adiantum scabrum* | RF | 0.950 | RF | 0.584 | RF | 0.832 | RF |
| *Adiantum sulphureum* | RF | 0.963 | RF | 0.668 | RF | 0.858 | RF |
| *Aextoxicon punctatum* | RF | 0.998 | RF | 0.950 | RF | 0.943 | RF |
| *Araucaria araucana* | GAM | 0.999 | RF | 0.992 | GAM | 0.989 | GAM |
| *Asplenium dareoides* | RF | 0.948 | RF | 0.697 | GBM | 0.820 | RF |
| *Asplenium monanthes* | GBM | 0.999 | GBM | 0.798 | RF | 0.830 | GBM |
| *Asplenium obtusatum var sphenoides* | GBM | 0.946 | RF | 0.697 | GBM | 0.841 | GBM |
| *Asplenium trilobum* | RF | 0.967 | RF | 0.626 | RF | 0.855 | RF |
| *Austrocedrus chilensis* | RF | 0.993 | RF | 0.923 | RF | 0.938 | RF |
| *Azara petiolaris* | RF | 0.994 | RF | 0.902 | RF | 0.957 | RF |
| *Blechnum arcuatum* | GBM | 0.904 | GBM | 0.625 | GBM | 0.720 | GBM |
| *Blechnum asperum* | RF | 0.998 | RF | 0.755 | FDA | 0.987 | RF |
| *Blechnum blechnoides* | RF | 0.977 | RF | 0.692 | RF | 0.919 | RF |
| *Blechnum chilense* | RF | 0.970 | RF | 0.736 | RF | 0.872 | RF |
| *Blechnum corralense* | RF | 0.964 | RF | 0.557 | RF | 0.924 | RF |
| *Blechnum hastatum* | RF | 0.980 | RF | 0.854 | RF | 0.902 | RF |
| *Blechnum magellanicum* | RF | 0.935 | RF | 0.552 | RF | 0.823 | RF |
| *Blechnum microphyllum* | RF | 0.943 | MARS | 0.575 | GBM | 0.791 | RF |
| *Blechnum mochaenum* | RF | 0.971 | RF | 0.757 | RF | 0.837 | RF |
| *Blechnum penna marina* | RF | 0.921 | RF | 0.552 | RF | 0.677 | RF |
| *Blepharocalyx cruckshanksii* | RF | 0.983 | RF | 0.864 | RF | 0.883 | RF |
| *Botrychium dusenii* | RF | 0.906 | RF | 0.598 | RF | 0.746 | RF |
| *Cheilanthes glauca* | RF | 0.958 | RF | 0.775 | RF | 0.782 | RF |
| *Cheilanthes hypoleuca* | RF | 0.961 | RF | 0.694 | RF | 0.826 | RF |
| *Cheilanthes mollis* | RF | 0.971 | RF | 0.730 | RF | 0.896 | RF |
| *Cryptocarya alba* | RF | 0.999 | RF | 0.979 | RF | 0.983 | RF |
| *Cryptogramma fumariifolia* | RF | 0.989 | GBM | 0.782 | RF | 0.963 | RF |
| *Cystopteris fragilis var apiiformis* | RF | 0.943 | RF | 0.639 | RF | 0.781 | RF |
| *Dennstaedtia glauca* | RF | 0.956 | RF | 0.450 | RF | 0.852 | RF |
| *Drimys andina* | RF | 0.989 | RF | 0.771 | GLM | 0.940 | RF |
| *Drimys winteri* | RF | 0.998 | RF | 0.941 | RF | 0.960 | RF |
| *Elaphoglossum gayanum* | RF | 0.994 | GBM | 0.676 | RF | 0.764 | RF |
| *Elaphoglossum mathewsii* | RF | 0.853 | RF | 0.698 | GBM | 0.713 | RF |
| *Elaphoglossum porteri* | RF | 0.999 | ANN | 0.999 | RF | 0.999 | RF |
| *Equisetum bogotense* | RF | 0.948 | RF | 0.705 | RF | 0.783 | RF |
| *Equisetum giganteum* | RF | 0.957 | RF | 0.678 | RF | 0.791 | RF |
| *Eucryphia cordifolia* | RF | 0.997 | RF | 0.971 | RF | 0.972 | RF |
| *Fitzroya cupressoides* | RF | 0.999 | RF | 0.977 | RF | 0.981 | RF |
| *Gevuina avellana* | RF | 0.998 | RF | 0.976 | RF | 0.972 | RF |
| *Gleichenia cryptocarpa* | GBM | 0.969 | RF | 0.654 | GBM | 0.880 | GBM |
| *Gleichenia litoralis* | RF | 0.960 | RF | 0.593 | RF | 0.862 | RF |
| *Gleichenia quadripartita* | RF | 0.917 | RF | 0.460 | RF | 0.739 | RF |
| *Gleichenia squamulosa* | RF | 0.965 | RF | 0.702 | RF | 0.874 | RF |
| *Gomortega keule* | RF | 0.999 | RF | 0.982 | RF | 0.998 | RF |
| *Grammitis magellanica* | RF | 0.928 | RF | 0.466 | GBM | 0.725 | RF |
| *Grammitis patagonica* | GBM | 0.973 | GBM | 0.395 | GBM | 0.953 | GBM |
| *Grammitis poeppigiana* | GBM | 0.948 | RF | 0.682 | GBM | 0.847 | GBM |
| *Hymenoglossum cruentum* | RF | 0.989 | RF | 0.740 | GBM | 0.889 | RF |
| *Hymenophyllum caudiculatum var productum* | RF | 0.954 | RF | 0.650 | RF | 0.834 | RF |
| *Hymenophyllum cuneatum* | RF | 0.975 | RF | 0.567 | RF | 0.842 | RF |
| *Hymenophyllum darwinii* | GBM | 0.932 | RF | 0.555 | GBM | 0.861 | GBM |
| *Hymenophyllum dentatum* | RF | 0.950 | RF | 0.702 | RF | 0.833 | RF |
| *Hymenophyllum dicranotrichum* | GBM | 0.925 | RF | 0.621 | GBM | 0.806 | GBM |
| *Hymenophyllum falklandicum* | RF | 0.909 | RF | 0.374 | GBM | 0.690 | RF |
| *Hymenophyllum ferrugineum* | RF | 0.958 | RF | 0.563 | GBM | 0.852 | RF |
| *Hymenophyllum fuciforme* | RF | 0.976 | RF | 0.607 | RF | 0.906 | RF |
| *Hymenophyllum krauseanum* | RF | 0.946 | RF | 0.586 | RF | 0.787 | RF |
| *Hymenophyllum nahuelhuapiense* | ANN | 0.997 | RF | 0.282 | GBM | 0.941 | ANN |
| *Hymenophyllum pectinatum* | RF | 0.949 | RF | 0.621 | RF | 0.790 | RF |
| *Hymenophyllum peltatum* | RF | 0.958 | RF | 0.585 | RF | 0.829 | RF |
| *Hymenophyllum plicatum* | RF | 0.950 | RF | 0.667 | RF | 0.867 | RF |
| *Hymenophyllum secundum* | RF | 0.926 | RF | 0.505 | RF | 0.755 | RF |
| *Hymenophyllum seselifolium* | RF | 0.939 | RF | 0.570 | GBM | 0.800 | RF |
| *Hymenophyllum tortuosum* | RF | 0.940 | RF | 0.573 | RF | 0.782 | RF |
| *Hymenophyllum tunbridgense* | RF | 0.919 | MARS | 0.567 | RF | 0.809 | RF |
| *Hymenophyllum umbratile* | RF | 0.979 | RF | 0.508 | RF | 0.957 | RF |
| *Hypolepis poeppigii* | RF | 0.942 | RF | 0.671 | GBM | 0.807 | RF |
| *Laurelia sempervirens* | RF | 0.998 | RF | 0.956 | RF | 0.960 | RF |
| *Laureliopsis philippiana* | RF | 0.999 | RF | 0.977 | RF | 0.961 | RF |
| *Lomatia hirsuta* | RF | 0.991 | RF | 0.924 | RF | 0.923 | RF |
| *Lophosoria quadripinnata* | GBM | 0.944 | RF | 0.575 | GBM | 0.768 | GBM |
| *Luma apiculata* | RF | 0.999 | RF | 0.966 | RF | 0.968 | RF |
| *Lycopodium alboffii* | RF | 0.972 | RF | 0.477 | RF | 0.741 | RF |
| *Lycopodium confertum* | GBM | 0.928 | RF | 0.167 | GBM | 0.776 | GBM |
| *Lycopodium gayanum* | RF | 0.937 | RF | 0.592 | RF | 0.774 | RF |
| *Lycopodium magellanicum* | RF | 0.941 | RF | 0.644 | GBM | 0.754 | RF |
| *Lycopodium paniculatum* | RF | 0.984 | RF | 0.750 | RF | 0.973 | RF |
| *Maytenus disticha* | RF | 0.986 | RF | 0.753 | RF | 0.924 | RF |
| *Megalastrum spectabile* | RF | 0.916 | RF | 0.610 | RF | 0.742 | RF |
| *Myrceugenia exsucca* | RF | 0.998 | RF | 0.951 | RF | 0.953 | RF |
| *Myrceugenia planipes* | RF | 0.986 | RF | 0.893 | RF | 0.937 | RF |
| *Nothofagus alessandrii* | RF | 0.999 | RF | 0.999 | RF | 0.999 | RF |
| *Nothofagus alpina* | RF | 0.999 | RF | 0.956 | RF | 0.974 | RF |
| *Nothofagus antarctica* | RF | 0.996 | RF | 0.929 | RF | 0.953 | RF |
| *Nothofagus betuloides* | RF | 0.994 | RF | 0.952 | RF | 0.967 | RF |
| *Nothofagus dombeyi* | RF | 0.999 | RF | 0.946 | RF | 0.961 | RF |
| *Nothofagus glauca* | RF | 0.999 | RF | 0.992 | RF | 0.992 | RF |
| *Nothofagus nitida* | RF | 0.999 | RF | 0.976 | RF | 0.991 | RF |
| *Nothofagus obliqua* | RF | 0.999 | RF | 0.974 | RF | 0.989 | RF |
| *Nothofagus pumilio* | RF | 0.994 | RF | 0.953 | RF | 0.958 | RF |
| *Pellaea myrtillifolia* | RF | 0.963 | RF | 0.510 | RF | 0.898 | RF |
| *Pellaea ternifolia* | GBM | 0.952 | RF | 0.426 | GBM | 0.843 | GBM |
| *Persea lingue* | RF | 0.999 | RF | 0.970 | RF | 0.973 | RF |
| *Philesia magellanica* | RF | 0.963 | RF | 0.623 | RF | 0.917 | RF |
| *Pilgerodendron uviferum* | RF | 0.996 | RF | 0.955 | RF | 0.960 | RF |
| *Pleopeltis macrocarpa* | RF | 0.990 | RF | 0.598 | RF | 0.987 | RF |
| *Pleurosorus papaverifolius* | RF | 0.959 | RF | 0.714 | RF | 0.867 | RF |
| *Podocarpus nubigenus* | RF | 0.990 | RF | 0.964 | RF | 0.956 | RF |
| *Podocarpus salignus* | RF | 0.983 | RF | 0.774 | RF | 0.930 | RF |
| *Polypodium feuillei* | RF | 0.967 | RF | 0.756 | RF | 0.782 | RF |
| *Polystichum andinum* | RF | 0.919 | RF | 0.490 | GBM | 0.815 | RF |
| *Polystichum chilense* | RF | 0.962 | RF | 0.726 | RF | 0.826 | RF |
| *Polystichum multifidum* | RF | 0.925 | RF | 0.390 | RF | 0.788 | RF |
| *Polystichum plicatum* | RF | 0.956 | RF | 0.713 | RF | 0.778 | RF |
| *Polystichum subintegerrimum* | GBM | 0.934 | RF | 0.386 | GBM | 0.824 | GBM |
| *Pteris chilensis* | RF | 0.970 | RF | 0.607 | RF | 0.885 | RF |
| *Pteris semiadnata* | RF | 0.966 | RF | 0.684 | RF | 0.777 | RF |
| *Rhaphithamnus spinosus* | RF | 0.997 | RF | 0.960 | RF | 0.957 | RF |
| *Rumohra adiantiformis* | RF | 0.971 | RF | 0.711 | RF | 0.892 | RF |
| *Schizaea fistulosa* | GBM | 0.985 | ANN | 0.747 | GBM | 0.951 | GBM |
| *Serpyllopsis caespitosa* | RF | 0.947 | RF | 0.523 | GBM | 0.923 | RF |
| *Tepualia stipularis* | RF | 0.996 | RF | 0.965 | RF | 0.974 | RF |
| *Thelypteris argentina* | RF | 0.987 | RF | 0.567 | RF | 0.815 | RF |
| *Trichomanes exsectum* | RF | 0.995 | RF | 0.765 | RF | 0.811 | RF |
| *Weinmannia trichosperma* | RF | 0.996 | RF | 0.964 | RF | 0.982 | RF |
