# Supplementary material for: In the Right Place at the Right Time: Habitat Representation in Protected Areas of South American Nothofagus-Dominated Plants after a Dispersal Constrained Climate Change Scenario
Source: PLoS One. 2015 Mar 18;10(3):e0119952. doi: 10.1371/journal.pone.0119952 (PMC4364909; doi:10.1371/journal.pone.0119952)
Supplement: S3 Table — (DOC) [file pone.0119952.s004.doc]

**Table S3.** **Assumptions used for each species in order to set MIGCLIM parameters^a^**.

| Species | Propagule dispersal syndrome | | | | SDD: Short distance dispersal^b^ (m) | LDD: Probability of long distance dispersal^c^ | Initial maturity age (years) | Optimal maturity age (years) |
| --- | --- | --- | --- | --- | --- | --- | --- | --- |
|  | Barochory | Zoochory | Anemochory | Hydrochory |  |  |  |  |
| *Adiantum chilense* |  |  | X |  | 900 | 0.2 | 2 | 5 |
| *Adiantum excisum* |  |  | X |  | 900 | 0.1 | 2 | 5 |
| *Adiantum gertrudis* |  |  | X |  | 450 | 0.05 | 2 | 5 |
| *Adiantum scabrum* |  |  | X |  | 900 | 0.1 | 2 | 5 |
| *Adiantum sulphureum* |  |  | X |  | 900 | 0.2 | 2 | 5 |
| *Aextoxicon punctatum* | X | X |  |  | 225 | 0.1 | 20 | 25 |
| *Araucaria araucana* | X | X |  |  | 45 | 0.1 | 25 | 45 |
| *Asplenium dareoides* |  |  | X |  | 900 | 0.1 | 2 | 4 |
| *Asplenium monanthes* |  |  | X |  | 450 | 0.05 | 2 | 5 |
| *Asplenium obtusatum var sphenoides* |  |  | X |  | 900 | 0.1 | 2 | 5 |
| *Asplenium trilobum* |  |  | X |  | 900 | 0.1 | 2 | 4 |
| *Austrocedrus chilensis* |  |  | X |  | 450 | 0.1 | 20 | 35 |
| *Azara petiolaris* |  | X |  |  | 45 | 0.1 | 10 | 15 |
| *Blechnum arcuatum* |  |  | X | X | 900 | 0.05 | 5 | 8 |
| *Blechnum asperum* |  |  | X |  | 900 | 0.1 | 2 | 5 |
| *Blechnum blechnoides* |  |  | X |  | 900 | 0.1 | 2 | 5 |
| *Blechnum chilense* |  |  | X |  | 900 | 0.2 | 5 | 8 |
| *Blechnum corralense* |  |  | X |  | 225 | 0.05 | 2 | 5 |
| *Blechnum hastatum* |  |  | X |  | 900 | 0.2 | 2 | 5 |
| *Blechnum magellanicum* |  |  | X |  | 900 | 0.1 | 5 | 8 |
| *Blechnum microphyllum* |  |  | X |  | 900 | 0.1 | 2 | 5 |
| *Blechnum mochaenum* |  |  | X |  | 900 | 0.1 | 2 | 5 |
| *Blechnum penna marina* |  |  | X |  | 900 | 0.1 | 2 | 5 |
| *Blepharocalyx cruckshanksii* |  | X |  |  | 90 | 0.1 | 15 | 25 |
| *Botrychium dusenii* |  |  | X |  | 900 | 0.1 | 2 | 5 |
| *Cheilanthes glauca* |  |  | X |  | 900 | 0.1 | 2 | 5 |
| *Cheilanthes hypoleuca* |  |  | X |  | 900 | 0.1 | 2 | 5 |
| *Cheilanthes mollis* |  |  | X |  | 900 | 0.1 | 2 | 5 |
| *Cryptocarya alba* | X | X |  |  | 450 | 0.2 | 20 | 25 |
| *Cryptogramma fumariifolia* |  |  | X |  | 900 | 0.1 | 2 | 5 |
| *Cystopteris fragilis var apiiformis* |  |  | X |  | 900 | 0.1 | 2 | 5 |
| *Dennstaedtia glauca* |  |  | X | X | 900 | 0.05 | 5 | 8 |
| *Drimys andina* |  | X |  |  | 45 | 0.1 | 10 | 15 |
| *Drimys winteri* |  | X |  |  | 90 | 0.2 | 10 | 20 |
| *Elaphoglossum gayanum* |  |  | X |  | 225 | 0.05 | 2 | 5 |
| *Elaphoglossum mathewsii* |  |  | X |  | 225 | 0.05 | 2 | 5 |
| *Elaphoglossum porteri* |  |  | X |  | 225 | 0.05 | 2 | 5 |
| *Equisetum bogotense* |  |  | X | X | 900 | 0.2 | 2 | 5 |
| *Equisetum giganteum* |  |  | X | X | 900 | 0.05 | 5 | 8 |
| *Eucryphia cordifolia* |  |  | X |  | 450 | 0.05 | 15 | 20 |
| *Fitzroya cupressoides* |  |  | X |  | 450 | 0.1 | 30 | 50 |
| *Gevuina avellana* | X | X |  |  | 90 | 0.1 | 10 | 20 |
| *Gleichenia cryptocarpa* |  |  | X |  | 900 | 0.1 | 2 | 5 |
| *Gleichenia litoralis* |  |  | X |  | 900 | 0.1 | 2 | 5 |
| *Gleichenia quadripartita* |  |  | X |  | 900 | 0.1 | 2 | 5 |
| *Gleichenia squamulosa* |  |  | X |  | 900 | 0.1 | 2 | 5 |
| *Gomortega keule* | X | X |  |  | 45 | 0 | 18 | 28 |
| *Grammitis magellanica* |  |  | X |  | 900 | 0.1 | 2 | 4 |
| *Grammitis patagonica* |  |  | X |  | 900 | 0.1 | 2 | 4 |
| *Grammitis poeppigiana* |  |  | X |  | 900 | 0.1 | 2 | 4 |
| *Hymenoglossum cruentum* |  |  | X | X | 450 | 0.05 | 2 | 4 |
| *Hymenophyllum caudiculatum var productum* |  |  | X | X | 450 | 0.05 | 2 | 4 |
| *Hymenophyllum cuneatum* |  |  | X | X | 450 | 0.05 | 2 | 4 |
| *Hymenophyllum darwinii* |  |  | X | X | 450 | 0.05 | 2 | 4 |
| *Hymenophyllum dentatum* |  |  | X | X | 450 | 0.05 | 2 | 4 |
| *Hymenophyllum dicranotrichum* |  |  | X | X | 450 | 0.05 | 2 | 4 |
| *Hymenophyllum falklandicum* |  |  | X | X | 450 | 0.05 | 2 | 4 |
| *Hymenophyllum ferrugineum* |  |  | X | X | 450 | 0.05 | 2 | 4 |
| *Hymenophyllum fuciforme* |  |  | X | X | 450 | 0.05 | 2 | 4 |
| *Hymenophyllum krauseanum* |  |  | X | X | 450 | 0.05 | 2 | 4 |
| *Hymenophyllum nahuelhuapiense* |  |  | X | X | 450 | 0.05 | 2 | 4 |
| *Hymenophyllum pectinatum* |  |  | X | X | 450 | 0.05 | 2 | 4 |
| *Hymenophyllum peltatum* |  |  | X | X | 450 | 0.05 | 2 | 4 |
| *Hymenophyllum plicatum* |  |  | X | X | 450 | 0.05 | 2 | 4 |
| *Hymenophyllum secundum* |  |  | X | X | 450 | 0.05 | 2 | 4 |
| *Hymenophyllum seselifolium* |  |  | X | X | 450 | 0.05 | 2 | 4 |
| *Hymenophyllum tortuosum* |  |  | X | X | 450 | 0.05 | 2 | 4 |
| *Hymenophyllum tunbridgense* |  |  | X | X | 450 | 0.05 | 2 | 4 |
| *Hymenophyllum umbratile* |  |  | X | X | 450 | 0.05 | 2 | 4 |
| *Hypolepis poeppigii* |  |  | X |  | 900 | 0.1 | 5 | 8 |
| *Laurelia sempervirens* |  |  | X |  | 900 | 0.1 | 20 | 25 |
| *Laureliopsis philippiana* |  |  | X |  | 900 | 0.1 | 20 | 30 |
| *Lomatia hirsuta* |  |  | X |  | 450 | 0.05 | 5 | 15 |
| *Lophosoria quadripinnata* |  |  | X |  | 900 | 0.2 | 5 | 8 |
| *Luma apiculata* |  | X |  |  | 90 | 0.1 | 5 | 15 |
| *Lycopodium alboffii* |  |  | X |  | 900 | 0.1 | 2 | 5 |
| *Lycopodium confertum* |  |  | X |  | 900 | 0.1 | 2 | 5 |
| *Lycopodium gayanum* |  |  | X |  | 900 | 0.1 | 2 | 5 |
| *Lycopodium magellanicum* |  |  | X |  | 900 | 0.1 | 2 | 5 |
| *Lycopodium paniculatum* |  |  | X |  | 900 | 0.1 | 2 | 5 |
| *Maytenus disticha* | X | X |  |  | 225 | 0.05 | 10 | 15 |
| *Megalastrum spectabile* |  |  | X |  | 900 | 0.1 | 5 | 8 |
| *Myrceugenia exsucca* |  | X |  |  | 45 | 0.1 | 15 | 20 |
| *Myrceugenia planipes* |  | X |  |  | 45 | 0.1 | 10 | 20 |
| *Nothofagus alessandrii* | X |  |  |  | 90 | 0.01 | 20 | 30 |
| *Nothofagus alpina* |  |  | X |  | 450 | 0.05 | 20 | 30 |
| *Nothofagus antarctica* |  |  | X |  | 450 | 0.05 | 20 | 30 |
| *Nothofagus betuloides* |  |  | X |  | 450 | 0.05 | 20 | 30 |
| *Nothofagus dombeyi* |  |  | X |  | 450 | 0.05 | 15 | 25 |
| *Nothofagus glauca* | X | X |  |  | 45 | 0.01 | 25 | 30 |
| *Nothofagus nitida* |  |  | X |  | 450 | 0.05 | 20 | 30 |
| *Nothofagus obliqua* |  |  | X |  | 450 | 0.05 | 15 | 25 |
| *Nothofagus pumilio* | X |  | X |  | 90 | 0.05 | 20 | 30 |
| *Pellaea myrtillifolia* |  |  | X |  | 900 | 0.1 | 2 | 5 |
| *Pellaea ternifolia* |  |  | X |  | 900 | 0.1 | 2 | 5 |
| *Persea lingue* | X | X |  |  | 450 | 0.2 | 10 | 20 |
| *Philesia magellanica* |  | X |  |  | 45 | 0.1 | 10 | 15 |
| *Pilgerodendron uviferum* |  |  | X |  | 450 | 0.1 | 25 | 45 |
| *Pleopeltis macrocarpa* |  |  | X |  | 450 | 0.05 | 2 | 5 |
| *Pleurosorus papaverifolius* |  |  | X |  | 900 | 0.1 | 2 | 5 |
| *Podocarpus nubigenus* | X | X |  |  | 45 | 0.01 | 20 | 40 |
| *Podocarpus salignus* | X | X |  |  | 45 | 0.01 | 10 | 20 |
| *Polypodium feuillei* |  |  | X |  | 900 | 0.1 | 2 | 5 |
| *Polystichum andinum* |  |  | X |  | 900 | 0.1 | 3 | 5 |
| *Polystichum chilense* |  |  | X |  | 900 | 0.1 | 3 | 5 |
| *Polystichum multifidum* |  |  | X |  | 900 | 0.1 | 3 | 5 |
| *Polystichum plicatum* |  |  | X |  | 900 | 0.1 | 3 | 5 |
| *Polystichum subintegerrimum* |  |  | X |  | 900 | 0.1 | 3 | 5 |
| *Pteris chilensis* |  |  | X |  | 900 | 0.1 | 2 | 5 |
| *Pteris semiadnata* |  |  | X |  | 900 | 0.05 | 5 | 8 |
| *Rhaphithamnus spinosus* | X | X |  |  | 45 | 0.2 | 5 | 15 |
| *Rumohra adiantiformis* |  |  | X |  | 900 | 0.05 | 5 | 8 |
| *Schizaea fistulosa* |  |  | X |  | 900 | 0.1 | 2 | 5 |
| *Serpyllopsis caespitosa* |  |  | X | X | 450 | 0.05 | 2 | 4 |
| *Tepualia stipularis* |  |  | X |  | 225 | 0.05 | 15 | 20 |
| *Thelypteris argentina* |  |  | X | X | 900 | 0.05 | 5 | 8 |
| *Trichomanes exsectum* |  |  | X | X | 225 | 0.05 | 2 | 4 |
| *Weinmannia trichosperma* |  |  | X |  | 900 | 0.1 | 20 | 30 |

^a^ MIGCLIM parameters were set following the available information on the studied species [51-65]. A dispersal kernel for propagules considered the short-distance dispersal (SDD) and the period reaching optimal maturity. The kernel of propagule production was assumed according to the period of years between the initial propagule production and the optimal maturity age, as predicted by a standard growth sigmoid function as follows; For 2 years: 0.4. For 3 years: 0.25, 0.7. For 5 years: 0.0659, 0.4974, 0.8357, 0.9549. For 10 years: 0.0046, 0.0659, 0.2521, 0.4974, 0.7019, 0.8357, 0.9130, 0.9549, 0.9769. For 15 years: 0.0007, 0.0118, 0.0659, 0.1889, 0.3600, 0.5345, 0.6811, 0.7902, 0.8656, 0.9153, 0.9472, 0.9672, 0.9798, 0.9875. For 20 years: 0.00009, 0.0021, 0.0167, 0.0659, 0.1639, 0.3004, 0.4493, 0.5873, 0.7019, 0.7902, 0.8550, 0.9010, 0.9330, 0.9549, 0.9698, 0.9798, 0.9865, 0.9910, 0.9940.

^b^ The short-distance dispersal (SDD) considered the distances according to the propagule dispersion syndrome: i) long-distance travelling propagule species, assumed to reach up to 900-1000 m; ii) medium-distance travelling propagule species, assumed to reach up to 400-500 m; iii) short-distance travelling propagule ground ferns, assumed to reach up to 200-250 m; iv) short-distance travelling propagule trees or shrubs, assumed to reach up to 80-100 m; and v) very short-distance travelling propagule species, assumed to reach up to 40-50 m [51-65].

^b^ The probability of long-distance dispersal (LDD) was assumed considering the abundance of natural populations and propagule dispersion syndromes: i) 0.2 or 20% for abundant ground fern species with anemochorous long-distance travelling spores and also for successfully zoochorous tree species which are very frequent; ii) 0.1 or 10% for medium-abundant fern species with anemochorous long-distance travelling spores, also for zoochorous medium-frequent tree and shrub species, and also for successfully anemochorous tree species; iii) 0.05 or 5% for scarce ground fern species, also for epiphytic fern species and also for anemochorous tree species which propagules are less able to reach long distances; iv) 0.01 or 1% for infrequent tree species with propagules with very low ability to reach long distances and v) 0 or 0% for species documented as very difficult or not able to establish away from their parent trees [51-65].
