# Supplementary material for: In the Right Place at the Right Time: Habitat Representation in Protected Areas of South American Nothofagus-Dominated Plants after a Dispersal Constrained Climate Change Scenario
Source: PLoS One. 2015 Mar 18;10(3):e0119952. doi: 10.1371/journal.pone.0119952 (PMC4364909; doi:10.1371/journal.pone.0119952)
Supplement: S4 Table — A full migration scenario is also included as a comparison for habitat sizes without using MIGCLIM. (DOC) [file pone.0119952.s005.doc]

**Table S4. Sensitivity analysis of MIGCLIM parameters SDD (short distance dispersal) and LDD (probability for long distance dispersal) and their effects on the habitat area size for all the studied species: a) by species group, and b) by each species.** A full migration scenario is also included as comparison for unconstrained future habitat sizes, without using MIGCLIM.

**a)**

| Change in MIGCLIM parameters | Effects on habitat size by species group (mean ± standard deviation) | | | | |
| --- | --- | --- | --- | --- | --- |
|  | *Nothofagus* | Co-dominant | Ground ferns | Epiphytic ferns | Total species |
| SDD x 25% | -0.6% ± 0.8% | -1.8% ± 2.2% | -5.9% ± 9.2% | -7.5% ± 8.8% | -4.9% ± 8% |
| SDD x 50% | -0.3% ± 0.6% | -0.8% ± 1% | -2.5% ± 3.6% | -3.2% ± 4.1% | -2.1% ± 3.3% |
| SDD x 200% | 0.1% ± 0.1% | 0.6% ± 0.7% | 0.6% ± 2.1% | 1.5% ± 2.1% | 0.8% ± 1.8% |
| LDD x 10% | 0% ± 0.1% | -0.3% ± 0.5% | -0.4% ± 0.6% | -0.1% ± 0.9% | -0.3% ± 0.7% |
| LDD x 25% | -0.1% ± 0.2% | -0.5% ± 0.5% | -0.6% ± 0.7% | -0.6% ± 0.8% | -0.5% ± 0.7% |
| LDD x 50% | 0% ± 0% | -0.3% ± 0.4% | -0.4% ± 0.4% | -0.4% ± 0.6% | -0.4% ± 0.5% |
| LDD x 200% | 0% ± 0.2% | 1.1% ± 1.1% | 1.6% ± 1.8% | 2.1% ± 3% | 1.5% ± 2% |
| LDD x 400% | 0.1% ± 0.1% | 1.1% ± 1% | 1.7% ± 1.8% | 2% ± 2.9% | 1.5% ± 2% |
| LDD x 1000% | 0.2% ± 0.1% | 1.9% ± 1.8% | 3.3% ± 3.7% | 4.2% ± 5.4% | 2.9% ± 3.9% |
| Full migration | 6.3% ± 12% | 12.2% ± 12% | 24.5% ± 30% | 34.1% ± 49.3% | 22.5% ± 32.8% |

b)

|  | Effect of different MIGCLIM parameters on habitat size | | | | | | | | | |
| --- | --- | --- | --- | --- | --- | --- | --- | --- | --- | --- |
| Species | SDD x 25% | SDD x 50% | SDD x 200% | LDD x 10% | LDD x 25% | LDD x 50% | LDD x 200% | LDD x 400% | LDD x 1000% | Full migration |
| *Adiantum chilense* | -4.8% | -2.4% | 0.0% | -0.8% | -0.7% | -0.4% | 1.8% | 1.9% | 2.3% | 13.8% |
| *Adiantum excisum* | -2.7% | -1.4% | -0.1% | -0.5% | -0.5% | -0.3% | 1.1% | 1.2% | 2.5% | 12.3% |
| *Adiantum gertrudis* | -0.2% | -0.1% | 0.0% | 0.0% | 0.0% | 0.0% | 0.1% | 0.1% | 0.2% | 2.5% |
| *Adiantum scabrum* | -0.8% | -0.2% | 0.0% | -0.1% | -0.1% | -0.1% | 0.5% | 0.5% | 1.0% | 3.1% |
| *Adiantum sulphureum* | -3.0% | -1.3% | 0.0% | -0.7% | -0.6% | -0.4% | 1.2% | 1.3% | 1.5% | 8.6% |
| *Aextoxicon punctatum* | -1.5% | -0.8% | 0.4% | -0.3% | -0.3% | -0.2% | 1.0% | 1.0% | 1.9% | 10.7% |
| *Araucaria araucana* | -0.4% | -0.2% | 0.1% | -0.2% | -0.2% | -0.1% | 0.4% | 0.4% | 0.7% | 8.5% |
| *Asplenium dareoides* | -2.4% | -1.0% | 0.0% | -0.4% | -0.3% | -0.2% | 0.9% | 0.9% | 1.6% | 8.5% |
| *Asplenium monanthes* | -12.2% | -4.5% | 2.3% | -0.5% | -0.3% | -0.2% | 1.8% | 1.8% | 4.9% | 27.5% |
| *Asplenium obtusatum var sphenoides* | -2.6% | -1.0% | 0.1% | -0.3% | -0.2% | -0.2% | 0.8% | 0.8% | 1.5% | 13.6% |
| *Asplenium trilobum* | -38.8% | -18.2% | -0.2% | -3.5% | -3.0% | -2.3% | 9.4% | 8.9% | 22.4% | 116.7% |
| *Austrocedrus chilensis* | -1.1% | -0.7% | 1.0% | -0.7% | -0.6% | -0.4% | 1.3% | 1.2% | 2.3% | 14.0% |
| *Azara petiolaris* | -2.2% | -1.2% | 1.1% | -0.8% | -0.7% | -0.5% | 1.8% | 1.7% | 3.0% | 9.5% |
| *Blechnum arcuatum* | -1.6% | -0.6% | 0.0% | -0.1% | -0.1% | -0.1% | 0.6% | 0.6% | 1.2% | 11.3% |
| *Blechnum asperum* | -19.2% | -8.3% | 0.1% | -1.2% | -1.2% | -0.8% | 3.3% | 3.3% | 6.3% | 46.7% |
| *Blechnum blechnoides* | -5.4% | -2.8% | 0.0% | -0.5% | -0.2% | -0.4% | 2.7% | 2.5% | 4.8% | 53.5% |
| *Blechnum chilense* | -1.2% | -0.5% | 0.0% | -0.5% | -0.4% | -0.3% | 0.8% | 0.7% | 0.9% | 11.4% |
| *Blechnum corralense* | -22.2% | -9.1% | 4.4% | -1.6% | -1.0% | -1.5% | 5.8% | 7.1% | 12.8% | 33.8% |
| *Blechnum hastatum* | -2.3% | -0.9% | 0.0% | -0.4% | -0.3% | -0.2% | 0.6% | 0.6% | 0.7% | 4.4% |
| *Blechnum magellanicum* | -3.4% | -1.4% | 0.0% | -0.8% | -0.7% | -0.6% | 2.3% | 2.3% | 4.4% | 28.8% |
| *Blechnum microphyllum* | -1.3% | -0.5% | -0.2% | -0.4% | -0.4% | -0.3% | 0.7% | 0.7% | 1.4% | 3.7% |
| *Blechnum mochaenum* | -4.9% | -1.9% | 0.1% | -0.8% | -0.6% | -0.4% | 1.9% | 1.9% | 3.5% | 33.5% |
| *Blechnum penna marina* | -3.1% | -1.2% | -0.1% | -0.4% | -0.4% | -0.3% | 0.9% | 0.9% | 1.8% | 7.4% |
| *Blepharocalyx cruckshanksii* | -3.7% | -1.7% | 1.3% | -1.6% | -1.2% | -0.8% | 3.2% | 3.3% | 6.0% | 22.3% |
| *Botrychium dusenii* | 0.0% | 0.0% | 0.0% | -0.2% | -0.2% | -0.2% | 0.6% | 0.5% | 0.9% | 6.2% |
| *Cheilanthes glauca* | -1.6% | -0.7% | -0.1% | -0.3% | -0.3% | -0.3% | 0.6% | 0.6% | 1.1% | 6.0% |
| *Cheilanthes hypoleuca* | -2.8% | -1.2% | -0.1% | -0.5% | -0.5% | -0.4% | 1.2% | 1.1% | 2.4% | 15.1% |
| *Cheilanthes mollis* | -8.5% | -3.9% | 0.0% | -0.7% | -0.6% | -0.5% | 1.6% | 1.6% | 3.8% | 16.8% |
| *Cryptocarya alba* | -0.4% | -0.2% | 0.1% | -0.2% | -0.2% | -0.1% | 0.5% | 0.4% | 0.5% | 8.6% |
| *Cryptogramma fumariifolia* | -0.8% | -0.1% | 0.2% | -0.1% | -0.1% | -0.1% | 0.7% | 0.6% | 1.1% | 4.4% |
| *Cystopteris fragilis var apiiformis* | -1.1% | -0.5% | 0.0% | -0.3% | -0.2% | -0.2% | 0.5% | 0.5% | 0.9% | 2.6% |
| *Dennstaedtia glauca* | -3.8% | -1.8% | -0.1% | -0.4% | -0.4% | -0.3% | 1.7% | 1.6% | 3.0% | 25.9% |
| *Drimys andina* | -3.0% | -1.5% | 1.0% | -1.8% | -1.5% | -1.0% | 2.6% | 2.6% | 3.4% | 7.4% |
| *Drimys winteri* | -1.0% | -0.5% | 0.5% | -0.9% | -0.7% | -0.4% | 1.3% | 1.3% | 1.5% | 8.9% |
| *Elaphoglossum gayanum* | -59.9% | -21.3% | 11.1% | -2.6% | -2.2% | -1.6% | 11.2% | 10.6% | 23.1% | 82.9% |
| *Elaphoglossum mathewsii* | -25.9% | -13.7% | 10.5% | -3.2% | -4.6% | -2.3% | 4.7% | 6.2% | 11.1% | 22.5% |
| *Elaphoglossum porteri* | -10.1% | -3.1% | 1.9% | -0.2% | -0.7% | -0.7% | 1.1% | 0.8% | 3.5% | 21.1% |
| *Equisetum bogotense* | -4.4% | -1.9% | 0.0% | -0.8% | -0.7% | -0.4% | 1.3% | 1.2% | 1.5% | 8.9% |
| *Equisetum giganteum* | -2.4% | -0.9% | 0.0% | -0.2% | -0.2% | -0.1% | 1.1% | 1.0% | 2.1% | 46.4% |
| *Eucryphia cordifolia* | -0.5% | -0.3% | 0.4% | -0.2% | -0.2% | -0.1% | 0.3% | 0.3% | 0.7% | 11.4% |
| *Fitzroya cupressoides* | -0.2% | 0.0% | 0.0% | -0.1% | -0.1% | -0.1% | 0.5% | 0.5% | 0.9% | 8.4% |
| *Gevuina avellana* | -1.3% | -0.6% | 0.4% | -0.5% | -0.4% | -0.2% | 0.9% | 1.0% | 1.7% | 7.2% |
| *Gleichenia cryptocarpa* | -0.4% | -0.2% | 0.0% | -0.3% | -0.3% | -0.1% | 0.5% | 0.5% | 0.8% | 4.4% |
| *Gleichenia litoralis* | -3.3% | -1.6% | -0.6% | -1.3% | -1.3% | -1.2% | 2.1% | 1.8% | 2.7% | 27.3% |
| *Gleichenia quadripartita* | -5.6% | -2.5% | 0.2% | -0.1% | -0.6% | -0.5% | 2.1% | 1.9% | 3.8% | 23.1% |
| *Gleichenia squamulosa* | -5.2% | -2.2% | -0.1% | -0.2% | -0.7% | -0.5% | 1.4% | 1.3% | 3.0% | 18.1% |
| *Gomortega keule* | -3.4% | -1.3% | 1.1% | 0.0% | -0.4% | 0.1% | -0.1% | -0.1% | 0.0% | 18.0% |
| *Grammitis magellanica* | -1.3% | -0.4% | 0.0% | 0.0% | -0.2% | -0.2% | 0.7% | 0.7% | 1.1% | 5.1% |
| *Grammitis patagonica* | -1.8% | -0.6% | 0.0% | 0.0% | -0.2% | -0.1% | 0.6% | 0.6% | 1.1% | 9.6% |
| *Grammitis poeppigiana* | -0.1% | 0.0% | 0.0% | 0.0% | -0.1% | 0.0% | 0.1% | 0.1% | 0.2% | 0.9% |
| *Hymenoglossum cruentum* | -3.2% | -1.5% | 1.0% | 0.0% | -0.2% | -0.2% | 0.7% | 0.7% | 1.8% | 20.1% |
| *Hymenophyllum caudiculatum var productum* | -8.3% | -4.3% | 1.9% | -1.0% | -1.2% | -1.2% | 0.9% | 0.7% | 2.5% | 9.8% |
| *Hymenophyllum cuneatum* | -15.4% | -6.0% | 3.2% | -0.1% | -0.7% | -0.5% | 2.6% | 2.7% | 5.1% | 45.0% |
| *Hymenophyllum darwinii* | -2.9% | -1.5% | 1.3% | 0.1% | -0.1% | -0.1% | 1.2% | 1.3% | 2.3% | 9.2% |
| *Hymenophyllum dentatum* | -2.5% | -1.0% | 0.6% | 0.1% | 0.0% | 0.0% | 0.6% | 0.6% | 1.1% | 9.0% |
| *Hymenophyllum dicranotrichum* | -2.6% | -0.9% | 0.6% | 0.0% | -0.1% | -0.1% | 0.5% | 0.5% | 1.0% | 9.5% |
| *Hymenophyllum falklandicum* | -6.2% | -2.7% | 1.9% | 0.0% | -0.3% | -0.3% | 1.7% | 1.8% | 3.6% | 25.3% |
| *Hymenophyllum ferrugineum* | -2.9% | -1.1% | 0.7% | 0.0% | -0.1% | -0.1% | 1.2% | 1.1% | 2.3% | 13.4% |
| *Hymenophyllum fuciforme* | -26.1% | -10.1% | 5.5% | -0.2% | -1.0% | -0.7% | 2.7% | 3.1% | 7.0% | 94.0% |
| *Hymenophyllum krauseanum* | -6.6% | -2.4% | 1.5% | 0.0% | -0.2% | -0.1% | 1.1% | 1.0% | 2.5% | 18.7% |
| *Hymenophyllum nahuelhuapiense* | -9.6% | -3.6% | 2.6% | 0.1% | 0.0% | 0.0% | 1.0% | 1.1% | 2.5% | 26.7% |
| *Hymenophyllum pectinatum* | -4.2% | -1.7% | 1.2% | 0.0% | -0.2% | -0.1% | 1.1% | 1.2% | 2.2% | 13.7% |
| *Hymenophyllum peltatum* | -6.2% | -2.3% | 1.0% | 0.0% | -0.5% | -0.3% | 1.3% | 1.3% | 2.7% | 23.0% |
| *Hymenophyllum plicatum* | -3.8% | -1.5% | 0.8% | 0.0% | -0.1% | -0.1% | 0.8% | 0.7% | 1.5% | 14.6% |
| *Hymenophyllum secundum* | -5.1% | -2.1% | 1.6% | 0.0% | -0.3% | -0.2% | 1.1% | 0.9% | 2.3% | 20.6% |
| *Hymenophyllum seselifolium* | -2.9% | -1.2% | 0.9% | 0.1% | -0.1% | -0.1% | 0.8% | 0.8% | 1.7% | 10.8% |
| *Hymenophyllum tortuosum* | -4.9% | -1.8% | 0.7% | 0.0% | -0.2% | -0.2% | 0.8% | 0.8% | 1.5% | 5.4% |
| *Hymenophyllum tunbridgense* | -9.4% | -4.7% | 2.2% | 2.9% | -3.1% | -2.2% | 14.2% | 13.8% | 20.0% | 226.8% |
| *Hymenophyllum umbratile* | -1.8% | -1.2% | 1.2% | 0.6% | -0.6% | -0.6% | 2.4% | 2.2% | 5.7% | 37.7% |
| *Hypolepis poeppigii* | -1.9% | -0.7% | -0.1% | 0.0% | -0.3% | -0.3% | 1.0% | 1.0% | 2.1% | 10.9% |
| *Laurelia sempervirens* | -0.6% | -0.1% | 0.0% | 0.0% | -0.2% | -0.2% | 1.1% | 1.1% | 2.0% | 9.4% |
| *Laureliopsis philippiana* | -0.3% | -0.1% | 0.0% | 0.0% | 0.0% | 0.0% | 0.2% | 0.2% | 0.4% | 5.2% |
| *Lomatia hirsuta* | -1.4% | -0.6% | 0.4% | 0.0% | -0.1% | 0.0% | 0.3% | 0.3% | 0.8% | 6.5% |
| *Lophosoria quadripinnata* | -0.3% | -0.1% | 0.0% | 0.0% | -0.1% | -0.1% | 0.2% | 0.2% | 0.3% | 3.7% |
| *Luma apiculata* | -0.7% | -0.4% | 0.3% | 0.0% | -0.2% | -0.1% | 0.5% | 0.5% | 0.9% | 4.6% |
| *Lycopodium alboffii* | -4.2% | -1.5% | -0.1% | -0.1% | -0.8% | -0.6% | 2.2% | 2.1% | 4.3% | 36.8% |
| *Lycopodium confertum* | -1.0% | -0.4% | 0.0% | 0.0% | -0.2% | -0.2% | 0.4% | 0.5% | 0.8% | 4.1% |
| *Lycopodium gayanum* | -13.1% | -4.6% | 0.1% | 0.2% | -0.6% | -0.3% | 2.2% | 2.3% | 4.4% | 60.7% |
| *Lycopodium magellanicum* | -3.7% | -1.7% | 0.0% | 0.0% | -0.3% | -0.2% | 0.9% | 0.9% | 1.9% | 7.6% |
| *Lycopodium paniculatum* | -6.5% | -2.8% | -0.2% | -0.2% | -0.7% | -0.6% | 1.7% | 1.8% | 3.7% | 18.2% |
| *Maytenus disticha* | -2.5% | -1.0% | 0.8% | 0.0% | -0.3% | -0.2% | 0.9% | 1.0% | 2.0% | 22.4% |
| *Megalastrum spectabile* | -3.4% | -1.4% | 0.0% | 0.0% | -0.6% | -0.4% | 1.9% | 1.9% | 3.7% | 19.4% |
| *Myrceugenia exsucca* | -3.3% | -1.6% | 1.3% | 0.0% | -1.5% | -0.9% | 2.9% | 2.8% | 4.7% | 13.5% |
| *Myrceugenia planipes* | -4.1% | -2.0% | 1.6% | 0.0% | -1.8% | -1.2% | 3.6% | 3.5% | 6.1% | 18.5% |
| *Nothofagus alessandrii* | -2.2% | -1.7% | 0.0% | -0.2% | -0.5% | 0.0% | -0.3% | -0.2% | 0.0% | 37.9% |
| *Nothofagus alpina* | -0.4% | -0.2% | 0.1% | 0.0% | 0.0% | 0.0% | 0.1% | 0.1% | 0.2% | 4.0% |
| *Nothofagus antarctica* | -0.4% | -0.2% | 0.1% | 0.0% | 0.0% | 0.0% | 0.1% | 0.1% | 0.2% | 3.1% |
| *Nothofagus betuloides* | -0.1% | -0.1% | 0.0% | 0.0% | 0.0% | 0.0% | 0.0% | 0.0% | 0.1% | 0.4% |
| *Nothofagus dombeyi* | -0.2% | 0.0% | 0.0% | 0.0% | 0.0% | 0.0% | 0.2% | 0.2% | 0.3% | 2.0% |
| *Nothofagus glauca* | -1.5% | -0.5% | 0.3% | 0.0% | 0.0% | -0.1% | 0.1% | 0.1% | 0.3% | 5.7% |
| *Nothofagus nitida* | -0.1% | -0.1% | 0.0% | 0.0% | 0.0% | 0.0% | 0.0% | 0.0% | 0.1% | 0.6% |
| *Nothofagus obliqua* | -0.1% | 0.0% | 0.0% | 0.0% | 0.0% | 0.0% | 0.1% | 0.1% | 0.2% | 1.9% |
| *Nothofagus pumilio* | -0.2% | -0.1% | 0.1% | 0.0% | 0.0% | 0.0% | 0.1% | 0.1% | 0.2% | 0.7% |
| *Pellaea myrtillifolia* | -1.1% | -0.5% | 0.0% | -0.1% | -0.5% | -0.3% | 0.6% | 0.6% | 1.1% | 6.0% |
| *Pellaea ternifolia* | -1.9% | -0.8% | 0.0% | 0.0% | -0.2% | -0.2% | 0.8% | 0.9% | 1.5% | 11.9% |
| *Persea lingue* | -0.6% | -0.3% | 0.2% | 0.0% | -0.4% | -0.2% | 0.7% | 0.7% | 0.8% | 5.0% |
| *Philesia magellanica* | -4.0% | -1.9% | 1.5% | 0.1% | -1.5% | -0.9% | 3.0% | 3.0% | 5.1% | 30.0% |
| *Pilgerodendron uviferum* | -0.1% | 0.0% | 0.0% | 0.0% | -0.1% | -0.1% | 0.4% | 0.4% | 0.6% | 4.7% |
| *Pleopeltis macrocarpa* | -23.9% | -11.7% | 10.1% | -0.3% | -1.7% | -1.3% | 4.8% | 4.9% | 11.1% | 111.0% |
| *Pleurosorus papaverifolius* | -2.4% | -1.1% | 0.0% | 0.1% | -0.2% | -0.1% | 0.9% | 0.9% | 1.9% | 9.1% |
| *Podocarpus nubigenus* | -0.9% | -0.4% | 0.3% | 0.0% | 0.0% | 0.0% | 0.1% | 0.1% | 0.3% | 2.3% |
| *Podocarpus salignus* | -11.0% | -4.6% | 2.9% | -0.1% | -0.4% | -0.4% | 1.0% | 0.8% | 2.4% | 61.2% |
| *Polypodium feuillei* | -5.3% | -2.2% | 0.0% | 0.0% | -0.5% | -0.4% | 1.5% | 1.7% | 3.2% | 18.3% |
| *Polystichum andinum* | -0.1% | -0.1% | 0.0% | -0.1% | -0.3% | -0.2% | 0.5% | 0.5% | 0.8% | 2.6% |
| *Polystichum chilense* | -4.2% | -1.8% | 0.1% | 0.0% | -0.5% | -0.3% | 1.7% | 1.7% | 3.5% | 29.3% |
| *Polystichum multifidum* | -3.9% | -1.7% | -0.2% | -0.1% | -0.8% | -0.6% | 1.5% | 1.5% | 3.2% | 20.9% |
| *Polystichum plicatum* | -0.3% | -0.1% | 0.0% | 0.0% | -0.1% | -0.1% | 0.3% | 0.4% | 0.6% | 2.2% |
| *Polystichum subintegerrimum* | -3.8% | -1.6% | 0.0% | -0.1% | -0.4% | -0.3% | 1.2% | 1.2% | 2.5% | 16.8% |
| *Pteris chilensis* | -13.1% | -5.9% | 0.1% | 0.1% | -1.4% | -1.0% | 4.6% | 4.2% | 8.7% | 64.5% |
| *Pteris semiadnata* | -12.5% | -5.6% | -0.2% | -0.1% | -1.1% | -0.8% | 3.9% | 4.0% | 8.4% | 132.5% |
| *Rhaphithamnus spinosus* | -0.9% | -0.4% | 0.4% | 0.0% | -0.9% | -0.5% | 1.2% | 1.2% | 1.3% | 7.2% |
| *Rumohra adiantiformis* | -2.7% | -1.1% | 0.0% | 0.0% | -0.2% | -0.1% | 0.9% | 0.8% | 2.0% | 29.7% |
| *Schizaea fistulosa* | -0.5% | -0.2% | 0.0% | 0.0% | -0.1% | -0.1% | 0.4% | 0.4% | 0.7% | 2.9% |
| *Serpyllopsis caespitosa* | -4.5% | -1.5% | 0.9% | 0.0% | -0.2% | -0.1% | 1.2% | 1.1% | 2.1% | 17.2% |
| *Tepualia stipularis* | -0.3% | -0.1% | 0.1% | 0.0% | 0.0% | 0.0% | 0.2% | 0.2% | 0.4% | 1.7% |
| *Thelypteris argentina* | -6.6% | -2.7% | 0.0% | 0.0% | -0.4% | -0.2% | 1.9% | 1.9% | 4.2% | 47.6% |
| *Trichomanes exsectum* | -11.3% | -5.4% | 1.7% | 0.1% | 0.0% | 0.1% | 1.3% | 1.2% | 3.6% | 162.0% |
| *Weinmannia trichosperma* | -0.1% | 0.0% | 0.0% | 0.0% | 0.0% | 0.0% | 0.1% | 0.1% | 0.2% | 1.2% |
