# Supplementary material for: In the Right Place at the Right Time: Habitat Representation in Protected Areas of South American Nothofagus-Dominated Plants after a Dispersal Constrained Climate Change Scenario
Source: PLoS One. 2015 Mar 18;10(3):e0119952. doi: 10.1371/journal.pone.0119952 (PMC4364909; doi:10.1371/journal.pone.0119952)
Supplement: S5 Table — (DOC) [file pone.0119952.s006.doc]

**Table S5. Modeled habitat area size (km^2^) under assessed scenarios: present and future (year 2050) with modeled dispersal constraints for each species and their representation under different conservation schemes in Chile.**

|  | Modeled Area (km^2^) under scenario: | | | | | | | | | | | | |
| --- | --- | --- | --- | --- | --- | --- | --- | --- | --- | --- | --- | --- | --- |
|  | Present | | | | | | Future under dispersal constraints | | | | | | Future without dispersal constraints |
| Species | NPA^a^ | PPA^b^ | Without protection | | | Total | NPA | PPA | Without protection | | | Total |  |
|  |  |  | SBN^c^ | SBR^d^ | Other^e^ |  |  |  | SBN | SBR | Other |  |  |
| *Adiantum chilense* | 2,679.1 | 579.4 | 5,313.0 | 8,081.1 | 57,341.8 | 73,994.3 | 4,565.2 | 1,364.7 | 7,505.0 | 9,456.1 | 76,338.6 | 99,229.6 | 109,451.6 |
| *Adiantum excisum* | 332.0 | 49.2 | 2,977.0 | 3,096.4 | 18,030.8 | 24,485.5 | 463.7 | 62.7 | 3,559.8 | 4,108.1 | 22,418.7 | 30,612.9 | 33,627.9 |
| *Adiantum gertrudis* | 89.4 | 25.3 | 2,775.7 | 1,286.3 | 5,107.0 | 9,283.6 | 96.7 | 23.1 | 2,374.0 | 1,266.0 | 4,983.5 | 8,743.4 | 8,978.8 |
| *Adiantum scabrum* | 994.2 | 95.6 | 4,632.0 | 7,360.3 | 21,855.3 | 34,937.4 | 1,913.7 | 164.9 | 5,723.3 | 9,199.8 | 29,118.9 | 46,120.6 | 47,211.2 |
| *Adiantum sulphureum* | 1,042.1 | 491.3 | 8,409.3 | 9,321.6 | 39,124.6 | 58,388.8 | 2,316.4 | 790.5 | 9,885.6 | 11,811.4 | 52,346.2 | 77,150.1 | 82,151.3 |
| *Aextoxicon punctatum* | 3,489.2 | 3,976.7 | 6,490.6 | 5,099.4 | 47,988.3 | 67,044.3 | 6,090.1 | 4,559.6 | 6,404.8 | 5,106.8 | 50,517.8 | 72,679.1 | 79,879.2 |
| *Araucaria araucana* | 2,829.1 | 112.7 | 286.2 | 2,279.7 | 11,437.0 | 16,944.7 | 2,291.1 | 205.0 | 304.2 | 913.0 | 5,773.8 | 9,487.1 | 10,923.6 |
| *Asplenium dareoides* | 9,082.6 | 3,636.9 | 3,954.4 | 4,735.4 | 41,818.2 | 63,227.6 | 11,539.2 | 5,361.3 | 4,981.1 | 5,550.7 | 57,528.7 | 84,961.0 | 90,362.0 |
| *Asplenium monanthes* | 396.1 | 177.7 | 60.4 | 505.1 | 3,334.0 | 4,473.3 | 863.4 | 623.4 | 9.3 | 654.6 | 4,366.7 | 6,517.5 | 7,745.9 |
| *Asplenium obtusatum* | 807.6 | 1,382.2 | 2,908.1 | 854.2 | 16,570.8 | 22,523.0 | 1,142.6 | 1,551.8 | 4,687.4 | 1,047.9 | 24,756.4 | 33,186.1 | 36,250.9 |
| *Asplenium trilobum* | 609.5 | 876.1 | 1,284.9 | 994.2 | 7,503.1 | 11,267.8 | 1,903.6 | 2,286.9 | 3,745.9 | 2,052.0 | 25,913.4 | 35,901.9 | 49,052.9 |
| *Austrocedrus chilensis* | 1,414.5 | 436.7 | 1,810.7 | 4,842.2 | 13,191.6 | 21,695.7 | 1,560.9 | 558.5 | 2,020.8 | 4,843.3 | 13,365.1 | 22,348.5 | 25,396.7 |
| *Azara petiolaris* | 250.2 | 80.5 | 2,238.8 | 3,071.6 | 4,341.5 | 9,982.7 | 344.9 | 88.9 | 2,188.6 | 2,993.6 | 4,369.3 | 9,985.3 | 10,934.6 |
| *Blechnum arcuatum* | 8,719.8 | 3,314.8 | 2,615.5 | 3,655.1 | 36,021.8 | 54,326.9 | 13,325.7 | 3,952.7 | 4,056.1 | 4,433.8 | 44,576.1 | 70,344.5 | 76,481.6 |
| *Blechnum asperum* | 1,172.9 | 709.5 | 660.1 | 1,384.4 | 11,238.6 | 15,165.5 | 3,382.8 | 1,800.2 | 1,827.2 | 2,280.6 | 28,498.1 | 37,788.9 | 44,878.5 |
| *Blechnum blechnoides* | 111.5 | 429.4 | 160.2 | 326.6 | 1,361.2 | 2,388.8 | 316.4 | 462.5 | 224.0 | 376.8 | 1,590.3 | 2,970.0 | 4,248.2 |
| *Blechnum chilense* | 6,981.7 | 2,894.0 | 7,391.7 | 9,051.2 | 70,529.9 | 96,848.5 | 10,081.4 | 4,097.7 | 9,242.7 | 10,352.0 | 88,490.1 | 122,263.8 | 133,277.1 |
| *Blechnum corralense* | 212.0 | 392.2 | 251.3 | 165.7 | 1,156.8 | 2,178.1 | 273.9 | 355.6 | 361.5 | 272.3 | 1,893.9 | 3,157.2 | 3,892.8 |
| *Blechnum hastatum* | 2,298.0 | 578.7 | 6,191.6 | 9,034.5 | 50,472.8 | 68,575.7 | 4,429.6 | 1,384.1 | 8,826.6 | 10,176.7 | 65,692.4 | 90,509.5 | 93,531.7 |
| *Blechnum magellanicum* | 8,065.1 | 593.0 | 2,505.8 | 4,117.9 | 23,973.2 | 39,255.0 | 8,933.6 | 1,083.5 | 3,126.6 | 4,697.8 | 28,630.9 | 46,472.4 | 57,770.2 |
| *Blechnum microphyllum* | 5,210.3 | 1,227.1 | 3,142.7 | 6,406.5 | 27,083.4 | 43,070.0 | 5,550.0 | 1,607.2 | 3,585.2 | 7,486.3 | 32,654.6 | 50,883.4 | 52,472.4 |
| *Blechnum mochaenum* | 1,050.5 | 448.2 | 728.7 | 1,572.4 | 8,151.1 | 11,951.0 | 2,156.3 | 327.0 | 1,196.7 | 2,196.1 | 12,038.8 | 17,914.9 | 21,922.9 |
| *Blechnum penna- marina* | 6,385.0 | 2,782.9 | 2,425.7 | 3,645.7 | 33,398.8 | 48,637.9 | 8,134.9 | 5,271.9 | 3,137.6 | 3,936.3 | 48,833.9 | 69,314.5 | 72,900.3 |
| *Blepharocalyx cruckshanksii* | 59.3 | 87.3 | 770.2 | 626.1 | 13,944.5 | 15,487.4 | 86.5 | 55.0 | 535.4 | 621.0 | 10,777.5 | 12,075.4 | 15,525.0 |
| *Botrychium dusenii* | 1,045.3 | 181.1 | 58.7 | 140.0 | 1,272.4 | 2,697.5 | 713.7 | 88.7 | 48.4 | 6.9 | 328.1 | 1,185.9 | 1,352.7 |
| *Cheilanthes glauca* | 2,850.7 | 194.3 | 6,195.0 | 8,978.1 | 25,975.8 | 44,193.9 | 3,653.9 | 276.9 | 6,780.5 | 9,616.6 | 31,032.6 | 51,360.5 | 54,028.4 |
| *Cheilanthes hypoleuca* | 466.5 | 163.0 | 4,247.3 | 5,409.5 | 14,344.8 | 24,631.1 | 431.4 | 126.5 | 5,207.2 | 4,694.9 | 18,690.4 | 29,150.3 | 32,857.8 |
| *Cheilanthes mollis* | 725.1 | 23.1 | 2,460.7 | 1,857.5 | 27,962.2 | 33,028.5 | 1,068.9 | 22.4 | 4,518.2 | 2,637.5 | 43,548.5 | 51,795.4 | 57,333.6 |
| *Cryptocarya alba* | 809.7 | 178.8 | 6,524.3 | 8,049.0 | 32,712.9 | 48,274.7 | 1,064.1 | 217.3 | 6,444.0 | 8,093.3 | 28,327.5 | 44,146.2 | 48,294.7 |
| *Cryptogramma fumariifolia* | 1,040.6 | 130.9 | 1,755.4 | 4,204.2 | 8,643.3 | 15,774.3 | 1,680.1 | 183.4 | 1,799.4 | 5,195.2 | 14,511.3 | 23,369.5 | 24,061.7 |
| *Cystopteris fragilis* | 8,662.4 | 2,987.7 | 10,212.2 | 10,867.8 | 69,396.2 | 102,126.2 | 7,997.3 | 4,361.8 | 11,309.9 | 12,358.6 | 78,729.6 | 114,757.3 | 117,430.9 |
| *Dennstaedtia glauca* | 437.5 | 141.1 | 3,580.2 | 3,555.2 | 6,614.4 | 14,328.2 | 577.6 | 158.0 | 4,845.4 | 4,596.4 | 11,002.7 | 21,180.1 | 24,892.5 |
| *Drimys andina* | 2,079.9 | 162.5 | 185.7 | 1,019.3 | 4,520.8 | 7,968.2 | 1,546.9 | 90.8 | 120.5 | 529.4 | 3,177.8 | 5,465.4 | 6,057.6 |
| *Drimys winteri* | 27,035.3 | 6,205.7 | 8,853.6 | 8,050.4 | 96,209.1 | 146,354.1 | 30,477.2 | 6,832.5 | 9,953.3 | 8,810.4 | 102,802.2 | 158,875.6 | 171,927.5 |
| *Elaphoglossum gayanum* | 490.9 | 372.7 | 502.8 | 201.6 | 2,742.8 | 4,310.8 | 733.0 | 936.9 | 993.9 | 914.6 | 12,003.1 | 15,581.4 | 19,153.4 |
| *Elaphoglossum mathewsii* | 182.5 | 1.3 | 9.7 | 2.0 | 228.8 | 424.3 | 308.4 | 0.0 | 0.0 | 5.1 | 313.4 | 626.9 | 722.5 |
| *Elaphoglossum porteri* | 422.5 | 0.0 | 0.0 | 0.0 | 143.5 | 566.0 | 620.0 | 0.0 | 0.0 | 0.0 | 294.9 | 914.8 | 1,034.4 |
| *Equisetum bogotense* | 4,027.1 | 1,417.5 | 6,089.5 | 9,618.9 | 46,388.0 | 67,541.0 | 6,137.0 | 2,273.4 | 8,150.6 | 10,904.9 | 70,203.5 | 97,669.3 | 103,679.4 |
| *Equisetum giganteum* | 215.7 | 38.9 | 2,577.9 | 1,716.7 | 7,416.9 | 11,966.1 | 494.8 | 39.6 | 2,731.0 | 2,279.3 | 9,225.2 | 14,769.8 | 20,324.1 |
| *Eucryphia cordifolia* | 2,527.3 | 2,302.8 | 4,965.1 | 2,637.5 | 32,407.5 | 44,840.3 | 3,939.6 | 3,267.4 | 4,645.5 | 2,150.6 | 28,404.2 | 42,407.4 | 47,512.2 |
| *Fitzroya cupressoides* | 4,160.7 | 2,611.0 | 2,768.4 | 590.0 | 10,077.0 | 20,207.2 | 3,027.2 | 1,517.5 | 2,020.0 | 109.2 | 6,575.0 | 13,249.0 | 14,946.5 |
| *Gevuina avellana* | 2,305.7 | 1,463.2 | 3,729.9 | 3,787.0 | 44,427.2 | 55,713.0 | 3,294.2 | 2,031.3 | 3,582.1 | 2,960.3 | 38,069.5 | 49,937.4 | 53,951.7 |
| *Gleichenia cryptocarpa* | 5,733.9 | 3,761.0 | 5,686.5 | 3,290.4 | 45,892.9 | 64,364.7 | 7,447.7 | 4,446.9 | 5,847.7 | 3,897.3 | 52,620.6 | 74,260.2 | 77,066.3 |
| *Gleichenia litoralis* | 192.5 | 629.9 | 312.0 | 128.4 | 1,064.8 | 2,327.6 | 42.8 | 231.4 | 138.4 | 76.9 | 567.1 | 1,056.6 | 1,693.1 |
| *Gleichenia quadripartita* | 6,929.1 | 1,615.9 | 1,202.0 | 1,496.7 | 17,621.2 | 28,864.9 | 9,340.5 | 2,489.7 | 1,505.1 | 1,333.5 | 24,162.0 | 38,830.8 | 45,492.3 |
| *Gleichenia squamulosa* | 969.9 | 823.8 | 2,605.0 | 1,467.1 | 20,409.6 | 26,275.4 | 2,121.9 | 2,260.8 | 4,859.0 | 1,716.4 | 34,922.7 | 45,880.9 | 50,646.4 |
| *Gomortega keule* | 37.0 | 7.6 | 140.6 | 81.7 | 1,148.9 | 1,415.8 | 28.2 | 7.6 | 84.5 | 57.6 | 815.0 | 992.9 | 1,247.3 |
| *Grammitis magellanica* | 33,065.3 | 4,601.8 | 5,257.5 | 5,152.5 | 72,497.8 | 120,574.9 | 40,675.5 | 6,260.7 | 7,530.4 | 6,552.9 | 93,056.6 | 154,076.1 | 160,228.2 |
| *Grammitis patagonica* | 8,236.9 | 1,490.7 | 594.3 | 695.8 | 37,372.8 | 48,390.5 | 9,115.6 | 1,500.6 | 1,118.7 | 734.1 | 52,971.7 | 65,440.7 | 70,072.9 |
| *Grammitis poeppigiana* | 8,345.5 | 2,072.9 | 1,458.1 | 2,039.7 | 16,095.2 | 30,011.4 | 4,840.5 | 991.7 | 469.5 | 690.2 | 8,484.9 | 15,476.8 | 15,761.0 |
| *Hymenoglossum cruentum* | 5,458.5 | 2,035.8 | 4,442.9 | 1,989.1 | 31,082.7 | 45,009.0 | 9,351.6 | 3,202.2 | 5,865.4 | 2,737.8 | 44,742.1 | 65,899.0 | 74,944.9 |
| *Hymenophyllum caudiculatum* | 2,346.0 | 1,480.1 | 1,804.7 | 1,286.3 | 16,746.7 | 23,663.7 | 3,930.5 | 3,258.6 | 4,332.7 | 2,030.9 | 27,725.1 | 41,277.8 | 43,591.4 |
| *Hymenophyllum cuneatum* | 3,255.9 | 815.2 | 890.6 | 771.2 | 11,989.3 | 17,722.3 | 5,485.2 | 1,790.7 | 2,939.6 | 1,422.1 | 25,310.9 | 36,948.4 | 44,925.5 |
| *Hymenophyllum darwinii* | 2,276.4 | 1,268.2 | 1,255.9 | 1,222.0 | 15,685.4 | 21,707.9 | 1,801.0 | 1,143.3 | 719.9 | 801.5 | 9,979.6 | 14,445.4 | 16,434.0 |
| *Hymenophyllum dentatum* | 5,853.6 | 3,782.7 | 3,971.3 | 3,068.7 | 35,539.6 | 52,215.9 | 7,872.3 | 4,408.6 | 5,541.2 | 3,682.7 | 45,865.1 | 67,369.9 | 72,062.2 |
| *Hymenophyllum dicranotrichum* | 6,067.3 | 3,295.8 | 4,817.3 | 2,229.5 | 36,916.6 | 53,326.5 | 9,792.5 | 4,100.9 | 5,520.4 | 2,701.5 | 46,405.1 | 68,520.5 | 73,604.8 |
| *Hymenophyllum falklandicum* | 4,741.8 | 1,026.9 | 986.7 | 1,939.0 | 11,653.2 | 20,347.5 | 5,014.9 | 1,466.5 | 1,158.9 | 1,732.1 | 12,860.1 | 22,232.5 | 27,371.4 |
| *Hymenophyllum ferrugineum* | 23,901.0 | 4,258.1 | 4,660.9 | 3,621.2 | 42,557.5 | 78,998.7 | 26,646.6 | 4,385.5 | 5,259.3 | 4,023.1 | 53,460.5 | 93,775.0 | 104,347.9 |
| *Hymenophyllum fuciforme* | 288.3 | 260.5 | 326.2 | 314.0 | 2,813.7 | 4,002.6 | 846.5 | 420.4 | 950.9 | 529.2 | 5,177.9 | 7,925.1 | 11,688.0 |
| *Hymenophyllum krauseanum* | 5,956.9 | 1,952.2 | 2,841.8 | 2,061.0 | 26,887.1 | 39,698.9 | 9,544.1 | 3,566.7 | 5,673.2 | 3,188.3 | 44,777.5 | 66,749.7 | 74,161.7 |
| *Hymenophyllum nahuelhuapiense* | 1,761.0 | 18.3 | 464.7 | 455.6 | 2,809.7 | 5,509.4 | 2,632.0 | 363.5 | 428.3 | 467.9 | 4,562.3 | 8,454.0 | 9,925.8 |
| *Hymenophyllum pectinatum* | 13,176.6 | 3,250.4 | 3,976.5 | 2,326.5 | 35,128.8 | 57,858.8 | 21,492.3 | 3,689.0 | 5,478.9 | 3,109.7 | 47,478.8 | 81,248.7 | 89,186.0 |
| *Hymenophyllum peltatum* | 3,375.1 | 2,328.3 | 2,391.3 | 1,485.5 | 15,271.5 | 24,851.7 | 2,576.2 | 2,539.8 | 4,080.1 | 1,579.0 | 18,072.4 | 28,847.6 | 34,569.1 |
| *Hymenophyllum plicatum* | 3,731.1 | 2,496.9 | 2,861.0 | 2,548.9 | 30,635.4 | 42,273.3 | 4,672.6 | 3,471.3 | 5,600.7 | 3,307.0 | 46,153.1 | 63,204.8 | 69,386.0 |
| *Hymenophyllum secundum* | 6,460.6 | 1,369.7 | 3,096.8 | 1,161.4 | 12,326.3 | 24,414.9 | 7,211.7 | 2,588.1 | 3,837.9 | 1,448.2 | 17,261.2 | 32,347.1 | 37,369.3 |
| *Hymenophyllum seselifolium* | 27,440.1 | 2,859.3 | 4,087.4 | 3,615.9 | 51,399.3 | 89,402.1 | 35,924.6 | 4,193.5 | 6,430.2 | 4,933.3 | 67,099.6 | 118,581.2 | 128,209.7 |
| *Hymenophyllum tortuosum* | 25,876.9 | 2,536.5 | 4,321.2 | 2,807.0 | 42,888.3 | 78,429.9 | 34,457.9 | 3,588.8 | 5,994.2 | 4,323.1 | 57,393.4 | 105,757.4 | 110,004.2 |
| *Hymenophyllum tunbridgense* | 3.4 | 0.0 | 0.7 | 2.7 | 374.7 | 381.6 | 27.5 | 0.0 | 0.0 | 27.1 | 373.2 | 427.9 | 1,293.2 |
| *Hymenophyllum umbratile* | 571.3 | 735.8 | 296.4 | 358.1 | 3,434.1 | 5,395.7 | 120.0 | 235.7 | 20.3 | 192.2 | 489.4 | 1,057.7 | 3,090.5 |
| *Hypolepis poeppigii* | 12,668.1 | 3,746.6 | 7,199.8 | 4,864.2 | 62,017.7 | 90,496.4 | 20,348.9 | 4,322.2 | 8,426.1 | 6,525.4 | 81,156.9 | 120,779.5 | 130,658.2 |
| *Laurelia sempervirens* | 1,378.2 | 474.4 | 2,595.8 | 4,543.5 | 31,009.2 | 40,001.0 | 2,169.7 | 648.3 | 2,907.2 | 5,168.7 | 32,679.0 | 43,572.9 | 47,341.1 |
| *Laureliopsis philippiana* | 11,018.7 | 4,696.9 | 5,966.0 | 2,605.2 | 48,652.0 | 72,938.8 | 11,474.2 | 4,799.2 | 5,566.7 | 2,004.7 | 42,938.5 | 66,783.4 | 70,608.3 |
| *Lomatia hirsuta* | 4,585.1 | 2,778.1 | 4,438.3 | 7,854.8 | 53,058.0 | 72,714.3 | 6,675.1 | 3,645.4 | 5,806.1 | 7,968.9 | 57,390.7 | 81,486.2 | 86,203.3 |
| *Lophosoria quadripinnata* | 17,013.8 | 5,170.1 | 6,959.3 | 5,482.3 | 82,903.6 | 117,529.2 | 19,883.6 | 5,351.6 | 7,180.1 | 5,882.5 | 87,829.5 | 126,127.2 | 130,442.9 |
| *Luma apiculata* | 11,558.0 | 5,236.0 | 7,905.1 | 8,516.3 | 79,894.4 | 113,109.7 | 13,116.7 | 5,422.4 | 8,595.4 | 8,646.1 | 82,924.6 | 118,705.2 | 123,890.9 |
| *Lycopodium alboffii* | 4,737.0 | 2,461.9 | 1,209.3 | 1,273.8 | 16,129.9 | 25,811.9 | 5,658.7 | 2,888.5 | 1,636.8 | 984.1 | 18,524.7 | 29,692.7 | 39,200.4 |
| *Lycopodium confertum* | 14,515.1 | 1,960.3 | 1,043.4 | 644.6 | 26,755.6 | 44,919.1 | 17,850.3 | 1,706.6 | 678.6 | 352.5 | 31,162.4 | 51,750.4 | 53,604.9 |
| *Lycopodium gayanum* | 822.4 | 507.5 | 764.5 | 303.9 | 3,299.5 | 5,697.7 | 837.4 | 663.1 | 1,090.9 | 588.3 | 7,031.6 | 10,211.3 | 13,668.9 |
| *Lycopodium magellanicum* | 17,951.9 | 5,144.2 | 2,504.2 | 5,673.8 | 45,607.6 | 76,881.8 | 22,577.5 | 6,330.8 | 3,771.2 | 6,354.3 | 62,283.0 | 101,316.9 | 107,167.0 |
| *Lycopodium paniculatum* | 3,304.0 | 1,874.3 | 2,662.2 | 1,135.9 | 15,099.5 | 24,075.9 | 4,362.4 | 2,434.9 | 2,691.3 | 2,293.9 | 25,951.8 | 37,734.3 | 42,120.7 |
| *Maytenus disticha* | 2,876.6 | 154.7 | 1,159.6 | 2,783.4 | 13,701.7 | 20,676.1 | 2,670.8 | 386.4 | 910.0 | 2,030.7 | 12,058.4 | 18,056.2 | 22,691.0 |
| *Megalastrum spectabile* | 4,273.0 | 2,000.5 | 2,663.7 | 3,250.7 | 32,211.3 | 44,399.2 | 6,056.4 | 3,423.1 | 5,239.6 | 4,283.0 | 47,677.1 | 66,679.2 | 75,299.0 |
| *Myrceugenia exsucca* | 861.6 | 1,434.5 | 5,292.5 | 3,213.9 | 42,866.3 | 53,668.9 | 1,706.3 | 2,373.5 | 6,431.7 | 3,965.2 | 45,241.3 | 59,718.0 | 66,965.9 |
| *Myrceugenia planipes* | 1,074.1 | 961.6 | 2,126.0 | 1,495.2 | 27,081.4 | 32,738.3 | 2,241.6 | 1,473.6 | 3,124.9 | 1,851.6 | 30,476.8 | 39,168.5 | 45,237.8 |
| *Nothofagus alessandrii* | 0.7 | 0.0 | 8.4 | 18.2 | 377.0 | 404.4 | 0.7 | 0.0 | 0.0 | 6.3 | 229.0 | 236.0 | 389.3 |
| *Nothofagus alpina* | 3,023.7 | 1,043.8 | 2,408.3 | 3,827.6 | 19,676.1 | 29,979.5 | 2,844.6 | 978.6 | 1,754.2 | 2,937.7 | 11,993.0 | 20,508.2 | 21,710.1 |
| *Nothofagus antarctica* | 44,886.1 | 7,413.6 | 7,711.5 | 8,159.8 | 134,355.8 | 202,526.7 | 44,978.9 | 7,383.5 | 7,770.4 | 6,816.0 | 123,225.9 | 190,174.7 | 196,451.3 |
| *Nothofagus betuloides* | 82,512.2 | 6,693.5 | 6,418.3 | 7,014.1 | 100,927.0 | 203,565.1 | 80,655.2 | 5,547.1 | 4,246.7 | 5,533.9 | 88,001.8 | 183,984.7 | 184,881.4 |
| *Nothofagus dombeyi* | 9,530.1 | 3,068.4 | 6,969.1 | 6,975.7 | 61,630.6 | 88,174.0 | 9,398.1 | 3,231.9 | 4,826.8 | 4,980.8 | 48,686.4 | 71,123.9 | 72,928.1 |
| *Nothofagus glauca* | 329.8 | 85.3 | 2,212.9 | 3,306.2 | 8,691.9 | 14,626.1 | 269.2 | 86.0 | 1,802.4 | 2,074.2 | 5,356.2 | 9,587.9 | 10,423.2 |
| *Nothofagus nitida* | 48,703.4 | 4,301.0 | 5,722.1 | 2,630.7 | 64,260.4 | 125,617.6 | 47,671.9 | 3,870.7 | 5,635.1 | 1,879.7 | 62,768.8 | 121,826.2 | 122,532.2 |
| *Nothofagus obliqua* | 3,779.1 | 1,745.2 | 5,348.5 | 7,462.3 | 48,852.9 | 67,188.0 | 4,228.8 | 1,923.3 | 5,222.1 | 6,755.4 | 43,971.7 | 62,101.3 | 63,388.0 |
| *Nothofagus pumilio* | 29,997.6 | 6,912.6 | 7,272.9 | 8,178.9 | 102,529.8 | 154,891.7 | 27,930.1 | 6,178.4 | 4,885.9 | 6,154.4 | 83,900.0 | 129,048.7 | 130,169.9 |
| *Pellaea myrtillifolia* | 21.2 | 23.7 | 1,176.4 | 1,227.0 | 11,584.2 | 14,032.5 | 71.5 | 20.9 | 1,460.2 | 1,606.7 | 12,572.9 | 15,732.1 | 16,570.6 |
| *Pellaea ternifolia* | 4,912.4 | 95.1 | 1,021.5 | 3,441.8 | 51,835.7 | 61,306.5 | 5,560.5 | 219.2 | 1,296.9 | 5,427.8 | 67,458.3 | 79,962.6 | 87,232.8 |
| *Persea lingue* | 1,378.2 | 888.7 | 1,888.0 | 3,613.5 | 36,604.1 | 44,372.5 | 2,175.7 | 1,468.9 | 2,543.6 | 3,498.0 | 33,530.1 | 43,216.3 | 45,436.5 |
| *Philesia magellanica* | 16,361.8 | 3,138.4 | 5,194.8 | 1,515.8 | 37,404.3 | 63,615.1 | 26,874.0 | 3718.5 | 5,494.7 | 1,599.2 | 47,790.0 | 85,476.5 | 104,584.1 |
| *Pilgerodendron uviferum* | 62,975.6 | 2,823.5 | 5,589.8 | 2,822.2 | 66,851.6 | 141,062.7 | 64,992.4 | 2766.2 | 4,891.7 | 2,233.3 | 66,416.6 | 141,300.1 | 147,910.2 |
| *Pleopeltis macrocarpa* | 874.8 | 610.4 | 1,261.5 | 1,957.6 | 15,913.6 | 20,617.8 | 2,244.1 | 685.9 | 1,610.8 | 4,100.6 | 33,480.0 | 42,121.4 | 65,009.5 |
| *Pleurosorus papaverifolius* | 528.0 | 143.3 | 4,560.0 | 5,088.9 | 12,868.6 | 23,188.9 | 724.1 | 215.5 | 5,263.2 | 5,765.1 | 15,828.1 | 27,796.0 | 29,912.0 |
| *Podocarpus nubigenus* | 63,971.4 | 4,353.6 | 6,107.6 | 3,569.5 | 72,795.7 | 150,797.7 | 64,767.1 | 3,813.9 | 5,628.3 | 2,577.2 | 67,753.8 | 144,540.3 | 147,939.0 |
| *Podocarpus salignus* | 594.6 | 377.9 | 1,201.5 | 1,405.3 | 11,171.7 | 14,751.0 | 1,037.4 | 523.6 | 1,612.8 | 2,393.4 | 11,148.7 | 16,715.9 | 25,737.6 |
| *Polypodium feuillei* | 1530.6 | 1,873.0 | 3,875.2 | 2,462.1 | 30,288.4 | 40,029.3 | 3,555.7 | 2,993.5 | 5,571.8 | 3,665.1 | 51,174.8 | 66,960.9 | 74,291.9 |
| *Polystichum andinum* | 6,127.2 | 1,171.5 | 2,278.0 | 3,653.1 | 10,050.5 | 23,280.3 | 3,379.0 | 498.5 | 1,566.3 | 1,963.0 | 5,016.9 | 12,423.7 | 13,018.5 |
| *Polystichum chilense* | 3,088.2 | 1,867.8 | 3,502.6 | 4,930.1 | 36,188.1 | 49,576.8 | 4,548.3 | 2,814.6 | 5,214.8 | 5,703.7 | 53,923.2 | 72,204.6 | 86,711.1 |
| *Polystichum multifidum* | 3,417.0 | 491.0 | 79.0 | 1,044.6 | 4,553.5 | 9,585.0 | 4,043.5 | 913.8 | 28.0 | 895.4 | 6,512.0 | 12,392.7 | 14,396.4 |
| *Polystichum plicatum* | 7,823.1 | 2,580.5 | 5,099.1 | 8,721.8 | 37,667.7 | 61,892.2 | 9,026.1 | 2,462.9 | 5,086.6 | 8,392.4 | 41,350.1 | 66,318.2 | 67,674.9 |
| *Polystichum subintegerrimum* | 1,879.9 | 1,141.9 | 1,726.0 | 2,817.8 | 30,418.6 | 37,984.2 | 4,475.8 | 2,277.2 | 3,275.5 | 3,282.4 | 43,069.7 | 56,380.7 | 62,778.0 |
| *Pteris chilensis* | 81.5 | 76.7 | 209.3 | 863.2 | 5,688.3 | 6,919.0 | 115.2 | 143.7 | 433.1 | 1,033.4 | 9,672.7 | 11,398.1 | 15,857.8 |
| *Pteris semiadnata* | 1,294.2 | 601.5 | 712.1 | 781.0 | 6,163.6 | 9,552.3 | 2,556.3 | 1,091.9 | 1,563.4 | 1,453.7 | 12,030.0 | 18,695.3 | 31,356.7 |
| *Rhaphithamnus spinosus* | 6,476.6 | 4,235.1 | 6,272.9 | 4,354.1 | 58,742.2 | 80,081.0 | 8,836.0 | 4,912.3 | 6,900.8 | 4,094.4 | 59,884.4 | 84,628.0 | 90,427.9 |
| *Rumohra adiantiformis* | 2,277.9 | 1,321.1 | 2,745.0 | 4,227.2 | 28,899.6 | 39,470.9 | 4,904.7 | 2,523.0 | 3,778.6 | 5,077.8 | 42,814.0 | 59,098.1 | 70,836.1 |
| *Schizaea fistulosa* | 7,520.0 | 1,647.1 | 4,755.1 | 670.0 | 22,430.4 | 37,022.5 | 11,943.6 | 1,684.8 | 4,817.1 | 660.6 | 23,302.4 | 42,408.5 | 43,472.8 |
| *Serpyllopsis caespitosa* | 6,209.3 | 1,373.8 | 3,928.2 | 1,313.9 | 21,770.4 | 34,595.5 | 8,840.4 | 2,364.3 | 4,813.4 | 1,637.8 | 28,908.4 | 46,564.2 | 52,503.2 |
| *Tepualia stipularis* | 58,386.6 | 3,857.7 | 6,050.5 | 1,645.1 | 63,980.2 | 133,920.0 | 54,307.4 | 3,384.3 | 5,988.9 | 897.5 | 59,723.1 | 124,301.1 | 126,563.4 |
| *Thelypteris argentina* | 933.7 | 41.4 | 5,698.0 | 3,706.0 | 34,751.9 | 45,130.9 | 1,620.1 | 82.2 | 8,614.6 | 6,374.5 | 65,701.5 | 82,392.9 | 103,872.3 |
| *Trichomanes exsectum* | 7.9 | 128.4 | 80.2 | 75.4 | 1,066.4 | 1,358.4 | 44.8 | 180.5 | 54.9 | 88.5 | 818.4 | 1,187.1 | 3,387.6 |
| *Weinmannia trichosperma* | 39,773.0 | 5,310.0 | 7,136.4 | 4,384.0 | 76,532.9 | 133,136.4 | 35,768.1 | 4,413.7 | 5,209.4 | 3,175.6 | 64,671.5 | 113,238.3 | 114,823.5 |

^a^ NPA: Representation under the national system of protected wild areas, managed by Chilean Government.

^b^ PPA: Representation under the private protected areas, managed by private owners in Chile.

^c^ SBN: Representation under the prioritized sites for biodiversity conservation, at a Chilean national level.

^d^ SBR: Representation under the prioritized sites for biodiversity conservation, at a scale of each Chilean administrative region.

^e^ Habitat areas without any protection scheme, also outside of any prioritized sites for biodiversity conservation in Chile.
