# Supplementary material for: In the Right Place at the Right Time: Habitat Representation in Protected Areas of South American Nothofagus-Dominated Plants after a Dispersal Constrained Climate Change Scenario
Source: PLoS One. 2015 Mar 18;10(3):e0119952. doi: 10.1371/journal.pone.0119952 (PMC4364909; doi:10.1371/journal.pone.0119952)
Supplement: S6 Table — (DOC) [file pone.0119952.s007.doc]

**Table S6. Habitat size comparisons among scenarios within species groups (obtained with paired t-tests).**

| Species group | Scenario | dispersal constrained future | unconstrained future |
| --- | --- | --- | --- |
| *Nothofagus* | present | t = -3.87, df = 8,  p-value = 0.004742 | t = -3.3695, df = 8,  p-value = 0.009793 |
|  | dispersal constrained future |  | t = -2.6222, df = 8,  p-value = 0.03055 |
| Co-dominant | present | t = -0.5985, df = 26,  p-value = 0.5547 | t = 2.1225, df = 26,  p-value = 0.04348 |
|  | dispersal constrained future |  | t = -7.9683, df = 26,  p-value = 1.906x10^-8^ |
| Ground ferns | present | t = 8.6654, df = 54,  p-value = 8.443x10^-12^ | t = 10.2558, df = 54,  p-value = 2.775x10^-14^ |
|  | dispersal constrained future |  | t = -9.9539, df = 54,  p-value = 8.051x10^-14^ |
| Epiphytic ferns | present | t = 6.0128, df = 26,  p-value = 2.382x10^-06^ | t = 6.9601, df = 26,  p-value = 2.171x10^-7^ |
|  | dispersal constrained future |  | t = -7.1138, df = 26,  p-value = 1.486x10^-7^ |
